# What is the evidence base to guide surgical treatment of infected hip prostheses? systematic review of longitudinal studies in unselected patients

**DOI:** 10.1186/1741-7015-10-18

**Published:** 2012-02-16

**Authors:** Andrew D Beswick, Karen T Elvers, Alison J Smith, Rachael Gooberman-Hill, Andrew Lovering, Ashley W Blom

**Affiliations:** 1 School of Clinical Sciences, University of Bristol, Musculoskeletal Research Unit, Southmead Hospital, Southmead Road, Bristol, BS10 5NB, UK; 2 Department of Microbiology, North Bristol NHS Trust, Bristol Centre for Antimicrobial Research and Enterprise, Southmead Hospital, Southmead Road, Bristol, BS9 3HU, UK

**Keywords:** hip replacement, hip arthroplasty, infection, revision, systematic review

## Abstract

**Background:**

Prosthetic joint infection is an uncommon but serious complication of hip replacement. There are two main surgical treatment options, with the choice largely based on the preference of the surgeon. Evidence is required regarding the comparative effectiveness of one-stage and two-stage revision to prevent reinfection after prosthetic joint infection.

**Methods:**

We conducted a systematic review to identify randomised controlled trials, systematic reviews and longitudinal studies in unselected patients with infection treated exclusively by one- or two-stage methods or by any method. The Embase, MEDLINE and Cochrane databases were searched up to March 2011. Reference lists were checked, and citations of key articles were identified by using the ISI Web of Science portal. Classification of studies and data extraction were performed independently by two reviewers. The outcome measure studied was reinfection within 2 years. Data were combined to produce pooled random-effects estimates using the Freeman-Tukey arc-sine transformation.

**Results:**

We identified 62 relevant studies comprising 4,197 patients. Regardless of treatment, the overall rate of reinfection after any treatment was 10.1% (95% CI = 8.2 to 12.0). In 11 studies comprising 1,225 patients with infected hip prostheses who underwent exclusively one-stage revision, the rate of reinfection was 8.6% (95% CI = 4.5 to 13.9). After two-stage revision exclusively in 28 studies comprising 1,188 patients, the rate of reinfection was 10.2% (95% CI = 7.7 to 12.9).

**Conclusion:**

Evidence of the relative effectiveness of one- and two-stage revision in preventing reinfection of hip prostheses is largely based on interpretation of longitudinal studies. There is no suggestion in the published studies that one- or two stage methods have different reinfection outcomes. Randomised trials are needed to establish optimum management strategies.

## Background

Hip replacement is widely used to treat pain associated with diseased or damaged joints. During the one-year period between April 2009 and March 2010, there were 71,021 primary hip replacement operations recorded in the National Joint Registry for England and Wales [1]. In the United States in 2006, the estimated number of hospital discharges after hip replacement was 231,000 [2], with demand predicted to increase substantially [3].

Deep prosthetic joint infection is an uncommon but serious complication of hip replacement [4]. Estimated incidences in UK populations are 0.45% to 0.57% [5,6], and the estimated incidence is 0.88% in the United States [7]. Infections that occur within 2 years of surgery are usually surgically acquired [8]. Surgical site infections have a very negative effect on patients. Typically, patients describe extreme pain, prolonged immobilisation, isolation and insecurity, as well as feelings of hopelessness [9]. Deep infection of hip prostheses puts patients at risk for these problems, and, if untreated, they can result in severe pain, persistent dislocation and death [10].

Conservative treatment with prosthesis retention may be considered in early infection if there is no evidence of prosthesis loosening or significant damage to soft tissue, as well as for infections that can be treated with antibiotics [8]. Typical rates of hip infection clearance of 89% [11] and 87% [12] have been reported recently. However, the need for long-term and possibly lifelong antibiotic treatment is acknowledged [12]. Treatment with prosthesis retention is not recorded in the National Joint Registry for England and Wales, but typically about 19% of prosthetic hip infections may be treated by using this method [13].

For the majority of patients with prosthetic hip infection, further major operations are required in an attempt to clear infection and reduce the need for joint excision or amputation. Surgical revision involves prosthesis removal and debridement with either immediate (one-stage) or delayed (two-stage) joint replacement or with excision (Girdlestone procedure). In England and Wales, about 500 hip revision procedures per year are required as a consequence of infection with treatment in one-stage revision (30%), two-stage revision (64%) or excision (6%) [1]. Two-stage revision is traditionally regarded as being more effective in treating infection, which probably explains the preponderance of two-stage revisions. The requirement of an extra hospital admission and a further major surgical procedure, however, means that a two-stage revision may cost 70% more than a one-stage revision [14].

Patients who undergo a two-stage revision are left without a joint between stages, and this is associated with pain and considerable disability. The period between stages is typically 2 to 12 months. In some patients, health status and infection severity may indicate that one revision method is appropriate [4]. In many cases where there is no clear advantage of one surgical method over another, however, the decision about treatment is made at the discretion of the surgeon. Using systematic review methods, we explored the evidence base supporting these decisions. We aimed to review evidence on the effectiveness of one-stage and two-stage revision in preventing reinfection after prosthetic hip infection.

## Methods

We used systematic review methods based on those described in the *Cochrane Handbook for Systematic Reviews of Interventions *[15] and in accordance with the Meta-analysis of Observational Studies in Epidemiology (MOOSE) proposal for reporting systematic reviews and meta-analysis of observational studies [16]. A MOOSE checklist is shown in Additional file 1, Table A1.

We searched the Embase, MEDLINE and Cochrane databases up to March 2011 for reviews, longitudinal studies and clinical trials. The search strategy used in MEDLINE is shown in Additional file 1, Table A2. The search included terms relating to hip replacement, infection and revision and focused on one- and two-stage surgeries. Thus, the search should have identified all studies in patients receiving contemporary surgical treatment options. We also searched reference lists and performed a citation search of key articles in the ISI Web of Science portal. No language restrictions were applied, which is an important consideration with the perceived international interest in treatment of infected hip prostheses.

Results of searches were stored in an EndNote X3/4 database (Thomson Reuters, New York, NY, USA). One reviewer (ADB) scanned titles and abstracts. Potentially relevant articles were acquired, and inclusion decisions were made separately, by two reviewers (ADB and KTE). Ultimately, decisions on inclusion were based on consensus.

We aimed to classify studies as follows: systematic reviews, randomised controlled trials or longitudinal studies in all patients with infection treated exclusively by one- or two-stage methods or by a variety of methods (including some by one- and some by two-stage). Studies excluded from this analysis reported case series of methods in selected patients (subsamples of patients who received revision in one or two stages or patients with a specific infection), had no outcomes by intention to treat, reported data for hip and knee combined or did not include patients with less than 2 years of follow-up.

To assess whether this selection strategy affected outcomes, we also analysed 2-year outcomes in any groups of patients who had received either one- or two-stage revision. We did not include studies where patients were treated only by joint excision with no prosthesis replacement.

Data were extracted in duplicate by two reviewers (ADB and KTE), with discrepancies resolved by discussion. Good practice in systematic reviews includes contact with authors to obtain additional information [15]. We did not contact authors, however, as many studies were published over 20 years ago and therefore we did not anticipate a good response.

The outcome we considered was reinfection within 2 years. No attempt was made to distinguish reinfection with the same or a new pathogen. As small case series are prone to selection issues, we report all studies in Additional file 1, Table A3, but discuss larger longitudinal studies (50 or more patients) in detail. To compare outcomes between methods, we created pooled random-effects estimates using the Freeman-Tukey arc-sine transformation to stabilise the variances.

## Results

The progress of the review is shown in Additional file 1, Figure A1. In our searches of databases, reference lists and citations, we identified 523 articles, 168 of which were subsequently found to be potentially relevant to this review. After reading the full papers, we included 66 articles in the review. The papers that we excluded mainly were selected case series of a particular surgical method rather than longitudinal studies of infected hip prostheses. The reasons for exclusion and references to the excluded studies are detailed in Additional file 1, Figure A1, and the references given in the Additional material.

The following studies were included: two systematic reviews comparing one- and two-stage methods in longitudinal studies [17,18], eleven studies (thirteen articles) of exclusively one-stage revisions (articles A1 to A13), twenty-eight studies (twenty-seven articles) of exclusively two-stage revisions (articles A14 to A40) and twenty-three studies (twenty-four articles) where treatments comprised one- or two-stage revisions and/or other surgical methods (articles A41 to A64). Two studies were randomised controlled trials evaluating use of antibiotic beads (article A30) or spacer (article A14), and groups were treated as separate studies. However, no randomised trials comparing one- and two-stage methods were identified.

The review article reported by Gallo and colleagues compared the outcomes of one-stage and two-stage revisions and excision arthroplasty in longitudinal studies [17]. The authors used systematic search methods but did not show the details of the 77 included studies published up to November 2006 in patients with hip and knee infections. The use of relative risks in their study indicates that the authors included studies where one- and two-stage methods were conducted in the same population. This approach does not address expected bias arising from selection of patients by health status and infection severity.

In their review, Wolf and colleagues reported an increased reinfection rate after one-stage revisions (12.3%) compared with two-stage revisions (6.5%) of infected total hip replacement [18]. In the studies they classified as two-stage revisions, however, more patients died. Searches up to May 2008 were limited to MEDLINE and publications in a major orthopaedic journal.

Longitudinal studies that we identified, regardless of size, are summarised in Additional file 1, Table A3. This includes descriptive information, our justifications for including the study as unselected (consecutive prosthetic hip infections) and the outcome as reflecting infection within 2 years of revision (if described). We also present an estimate of the 2-year reinfection rate in each study. Studies including 50 or more patients are summarised in Table 1.

**Table 1:** Characteristics of included studies with 50 or more patients

Study Country Year of study	Patient treatments Number of participants Mean age (% men)	Exclusive surgical method Treatment	Overall follow-up Outcomes Deaths and losses to follow-up	*N *(%) reinfection at 2 years Details
One-stage				
Buchholz *et al.*, 1981 [19] Germany 1968 to 1977	Hip arthroplasty for OA (95%), others (5%) *N *= 640 58.8 years (39.7%)	"Patients with deep infection involving arthroplasties of the hip" (p. 344). Antibiotic-loaded cement	52 months Need for further exchange, reinfection 90 deaths	99 (15.5%) Reoperation due to "bad" outcome plus other infection-related outcomes
Loty *et al.*, 1992 [20] France 1980 to 1988	THR *N *= 90 65.7 years (not specified)	"We usually manage infected total hip replacements by a one stage revision" (p. 330). Systemic antibiotics and antibiotic-loaded cement	47 months ± 29 months Reinfection, other failure, Merle d'Aubigné-Postel score Four deaths, seven lost to follow-up	8 (8.9%) Infections occurred between 6 and 24 months
Miley *et al.*, 1982 [21] USA 1969 to 1979	Hip surgery for fracture dislocation (47%), OA (36%), others (17%) *N *= 100 (101 hips) Men 56.2 years, women 59 years (53%)	"The operating surgeon must be prepared to perform either of these operations (1-stage or Girdlestone), depending on the surgical findings and medical work-up" (p79). Intensive multiple-drug antimicrobial programme	48.5 months, minimum 32 months Grading system focusing on quality of life and hip function. Grade IV outcome: drainage, constant pain, further surgery suggested 11 deaths	Eight hips (7.9%) Grade IV outcome No information on reinfection within 2 years
Raut *et al.*, 1995 [22] Wroblewski, 1986 [24] UK 1979 to 1990	Cemented primary THR (63%), revision THR (37%) *N *= 183 64.5 years (48%)	"All infected arthroplasties during this period were treated with 1-stage reimplantation, irrespective of the organism that was cultured, unless the patient had poor quality bone stock." (p. 202. Wroblewski, 1986 [24]) Antibiotic-loaded cement	93 months, range 24 to 164 months Persistent infection, Merle d'Aubigné-Postel score (pain, function and movement), radiology, re-revision, complications. 14 deaths (0 in year 1)	6 (3.3%) Reinfection in first 2 years
Schneider, 1989 [23] Switzerland 1973 to 1988	Hip implants *N *= 72 (excluding 13 treated with irrigation alone) Not specified	"Between 1980 and 1988, out of 42 deep infections...." (p. 527) Data also included from 1973 onwards Joint irrigation preceding revision	Follow-up interval not specified. Reinfection (bad outcome)	19 (26.4%), rate from 1980 (16.1%) No information on reinfection within 2 years
Two-stage				
Chen *et al.*, 2009 [25] Taiwan 1993 to 2005	THR (excluding patients with proximal femoral allograft reconstruction) *N *= 57 51.5 years (72%)	"[A]ccording to the protocol for short-term parenteral antibiotics therapy at this institution...." (p. 189). Interim antibiotic-impregnated cement beads	67.2 months Reinfection, Harris hip scores, radiological examinations Five deaths and five lost to follow-up	7 (12.3%) Reinfection in seven patients after first stage described in text
Fitzgerald and Jones, 1985 [26] USA 1969 to 1979	Hip implant *N *= 131 61 years (50%)	"[D]elayed reconstruction in 131 patients who had an infection after a previous total hip arthroplasty...." (p.828. McDonald et al. 1989 [32]). Intensive Cemented reconstruction with no added antibiotic	49 months; range 2 to 9 years Reinfection	All hip implants 11 (8.4%) Reinfection up to 429 days
McDonald *et al.*, 1989 [32] 1969 to 1985	Specifically THR for OA (69%), fracture (13%), other (18%) *N *= 81 (including additional 13 patients) 60.0 years (53%)		5.5 years, range 2.0 to 13.6 years Reinfection, complications	THR only 6 (7.4%) estimated from survival curve
Haddad *et al.*, 2000 [27] UK 1988 to 1992	THR for OA (72%), other (28%) *N *= 50 60 years (46%)	"[C]onsecutive patients all of whom were referred with an infected total hip replacement and treated using a standardised protocol" (p. 689). Antibiotic-loaded beads and cement ball Uncemented	5.8 years, range 2 to 8.7 years Reinfection, Harris hip score, radiological outcome, complications Two deaths	4 (8%) No information on reinfection within 2 years
Hsieh *et al.*, 2009 [28] Taiwan 2002 to 2005	Prosthetic hip *N *= 99 61 years (61%)	"The use of an ALCS in SEA for PHI has been a routine practice in our institution" (p. 93). Antibiotic-loaded cement spacer	43 months, range 24 to 60 months Reinfection, Merle d'Aubigné-Postel score (only in comparisons of antibiotic strategies), radiographic results Three deaths, five lost to follow-up	8 (8.1%) Reinfection between stages
Romanò *et al.*, 2010 [29] Italy 2000 to 2007	Hip prostheses *N *= 102 58 years (34%)	"102 consecutive patients underwent two-stage revision of septic hip replacement" (p. 26). Long-stem or short-stem preformed antibiotic-loaded cement spacers Cementless	48 months Reinfection, Harris hip score (only in comparisons of antibiotic strategies) Three deaths, nine lost to follow-up	5 (4.9%) Reinfection within 3 years (including between stages)
Stockley *et al.*, 2008 [30] UK 1991 to 2004	THR for OA (60%), posttraumatic arthritis (18%), others (23%) *N *= 114 64 years (55%)	"[C]onsecutive patients with microbiologically-proven deep chronic infection of the hip were managed by a two-stage exchange procedure" (p.145). Antibiotic-loaded cement beads	74 months, range 2 to 175 months Reinfection	9 (7.9%) Reinfection within 1 year
Toulson *et al.*, 2009 [31] USA 1989 to 2003	THR *N *= 132 54.7 years (59%) in patients "who completed the entire protocol" (p.1054)	"All 132 cases of infected THAs treated at our institution...." (p.1052). Spacer containing antibiotic impregnated cement used in 67%	64.8 months, range 24 to 203 months; eight patients followed for only an average of 7.2 months Reinfection, Harris hip score (mean only) 34 deaths (2 with no infection information), 8 lost to follow-up	11 (8.3%) Three new infections within 24 months, four infections not eradicated, four patients who died had infections
Combination of methods				
De Man *et al.*, 2011 [33] Switzerland 1985 to 2004	THR *N *= 79 hips 70 years (57%) in patients followed up	One-stage (*n *= 24), two-stage (*n *= 55) Some patients had spacers between stages	Mean 3.8 years (SD 2.2) Reinfection, Harris hip score including limping and walking, radiographic outcome Seven lost to follow-up	Overall (*n *= 2, 2.5%) One-stage (*n *= 1, 4.5%) Two-stage (*n *= 1, 2.0%)
Elson, 1993 [34] UK Not specified	THR *N *= 296 (definite or possible infection) Not specified	One-stage (*n *= 235), two-stage (*n *= 61) Antibiotic-loaded cement pellets used in two-stage method	Not specified Reinfection, mechanical survival, radiological outcome	Overall (*n *= 36, 12.2%) One-stage (*n *= 33, 14.0%) Two-stage (*n *= 3, 4.9%) Time of definite or possible reinfection (mean 25 months, range 1 to 68 months)
Ketterl *et al.*, 1988 [35] Germany 1976 to 1986	THR *N *= 207 69 years (42%)	One-stage (*n *= 21), two-stage (*n *= 161), no reimplantation (*n *= 25) Gentamicin cement	32 months Reinfection, function	Overall (*n *= 24, 11.6%) One-stage (*n *= 7, 33.3%) Two-stage (*n *= 17, 10.6%) Time of reinfection unclear
Ladero Morales *et al.*, 1999 [36] Spain 1985 to 1995	THR *N *= 62 (another 12 with nonsurgical treatment) 74 years (53%)	One-stage (*n *= 2), two-stage (*n *= 37), resection-only (*n *= 23) Some treated after introduction of perioperative antibiotic protocol	4.8 years Reinfection, Merle d'Aubigné-Postel score, clinical and functional outcome (pain, mobility, gait)	Overall (*n *= 3, 4.8%) One-stage (*n *= 0, 0%) Two-stage (*n *= 3, 8.1%) Time of reinfection unclear
Lecuire *et al.*, 1999 [37] France 1982 to 1997	THR *N *= 57 70.6 years	One-stage (*n *= 16), two-stage (*n *= 41) Uncemented	6.6 years Reinfection, Merle d'Aubigné-Postel scale, Harris hip score	Overall (*n *= 2, 3.5%) One-stage (*n *= 1, 6.3%) Two-stage (*n *= 1, 2.4%) Time of reinfection unclear
Oussedik *et al.*, 2010 [38] UK 1999 to 2002	THR *N *= 50 65 years (42%)	One-stage (*n *= 11), two-stage (*n *= 39) Antibiotic-loaded spacer used in two-stage Gentamicin-loaded cement used in one-stage	6.8 years, range 5.5 to 8.8 years Reinfection, Harris hip score, VAS satisfaction	Overall (*n *= 2, 4.0%) One-stage (*n *= 0, 0%) Two-stage (*n *= 2, 5.1%) No information on reinfection within 2 years
Sanzén *et al.*, 1988 [39] Carlsson *et al.*, 1978 [42] Sweden 1974 to 1981	THR for OA (74%), congenital dislocation (8%), fracture (8%), others (10%) *N *= 108 (110 hips) 64 years (53%)	One-stage (*n *= 78 hips), 2-stage (*n *= 32 hips) In 44% of two-stage procedures, gentamicin-loaded PMMA beads were used Gentamicin-loaded cement	71 months, range 24 to 117 months Reinfection, loosening, function Eight deaths within 24 months	Overall (*n *= 22, 20.0%) One-stage (*n *= 17, 21.8%) Two-stage (*n *= 8, 25.0%) At least 22 of 25 reinfections in first year
Vielpeau and Lortat-Jacob, 2002 [40] France Up to December 1998	THR *N *= 458 (including acetabular or femoral revision only, excluding methods with retention of components) No age or sex details	One-stage (*n *= 127), two-stage (*n *= 222), resection (*n *= 81) Antibiotic cement (*n *= 249), no antibiotic cement (*n *= 100) in one or two stages	Median 3 years, 81.5% followed for minimum of 2 years Reinfection, complications	Overall (*n *= 72, 15.7%) One-stage (*n *= 15, 11.8%) Two-stage (*n *= 33, 14.9%)

ALCS, antibiotic-loaded cement spacer; OA, osteoarthritis; PHI, prosthetic hip infection; PMMA, polymethylmethacrylate; SEA, staged exchange arthroplasty; THA, total hip arthroplasty; THR, total hip replacement; VAS, Visual Analogue Scale.

The studies we included reported data from infected hips identified over extended periods with a mean of 8.5 years (range 3 to 17 years) for studies of exclusively one- or two-stage revisions. Generally, information on reinfection was clear, and, in some articles, data on individual patients was provided. Where possible, we used data on reinfection within 2 years of treatment, but this was not always the case.

### One-stage revisions

Reinfection rates in the five studies with over 50 patients (range 72 to 640) were 15.5% [19], 8.9% [20], 7.9% [21], 3.3% [22] and 26.4% [23]. In three of the studies, the use of antibiotic-loaded bone cement was studied [19,20,22].

An early series described by Buchholz and colleagues included 640 revisions of infected hip prostheses treated exclusively by a one-stage procedure [19]. The authors reported "bad results," which included failure to eradicate infection and achievement of stable joint replacement. The authors reported a success rate of 77% after one-stage revision, with a stable, infection-free joint achieved in 90% of patients after further revision operations. By estimating an outcome related directly to infection, we calculated that 15.5% of patients had early reinfection. This may represent an overestimate, however, because we cannot be certain that all of these reinfections occurred within 2 years or that all second exchange operations were performed as a consequence of reinfection.

In 90 patients followed up by Loty and colleagues, the rate of reinfection within 2 years was 8.9% [20]. This may have been an underestimate, however, as seven patients (7.8%) were lost to 2-year follow-up. Long-term function measured by the Merle d'Aubigné-Postel score was good or very good in 79% of patients followed up.

Miley and colleagues reported a study of 100 patients with 101 infections after hip surgery and reported outcomes from grades I to IV [21]. Grade IV, reflecting drainage, constant pain and the suggestion of a need for further surgery, was recorded in 7.9% of hips. The incidence of reinfection within 2 years was not specified. Raut and colleagues reported on a cohort of 183 patients, among whom the reinfection was 3.3% within 2 years of revision of infected hip replacement [22,24]. No pain, as measured by the Merle d'Aubigné-Postel score, was reported by 69% of patients at last follow-up. Thirteen percent of patients were dissatisfied with their outcomes.

Schneider followed up 72 patients with infected hip implants [23]. A bad outcome, mainly reinfection, was reported in 26.4% of patients, but this may have included those with reinfection after 2 years. The author reported that the reinfection rate in patients with infection identified from 1980 onwards was 16.1%.

### Two-stage revisions

Seven studies of 50 or more patients reported outcomes of two-stage revisions of infected hip prostheses. The reinfection rates reported were 12.3% [25], 8.4% [26], 8.0% [27], 8.1% [28], 4.9% [29], 7.9% [30] and 8.3% [31]. Antibiotic-loaded cement beads [25,27,30] or spacers [28,29,31] were used between stages in six studies.

Chen and colleagues reported a 2-year reinfection rate of 12.3% after treatment of infected hip replacement [25]. This was mainly restricted to the period between stages, with repeated debridements used to achieve a lower proportion of patients with infection at 2 years (3.5%). An excellent or good functional recovery according to the Harris hip score was recorded in 74% of patients. In a study of 131 patients with infected hip implants, Fitzgerald and Jones reported a reinfection rate within 14 months of 8.4% [26,32]. Haddad and colleagues followed up 50 patients with infected hip replacement and reported reinfection in 8.0% of them [27]. It was not clear whether these reinfections occurred within 2 years of hip replacement. A good or excellent functional outcome according to the Harris hip score was achieved by 78% of patients.

In a study of 99 patients with infected hip prostheses, Hsieh and colleagues reported reinfection between stages in 8.1% of patients [28]. Romanò and colleagues followed up 102 patients with infected hip prostheses [29]. Within 3 years of revision, 4.9% of patients had reinfection. A further 8.8% of patients were lost to follow-up. In a study of 114 patients with infected total hip replacement, Stockley and colleagues reported reinfection within 1 year in 7.9% of patients [30]. Toulson and colleagues reported reinfection in 8.3% of patients within 2 years of total hip replacement [31].

### Studies with combination of one-stage, two-stage and excision procedures

In addition to the studies of specifically one-stage and two-stage revisions, searches identified studies in which patients with infected hip prostheses were followed up after treatment by any one of one-stage, two-stage or excision methods. In eight studies of 50 or more patients, the rates of reinfection were 2.5% [33], 12.2% [34], 11.6% [35], 4.87% [36], 3.5% [37], 4.0% [38], 20.0% [39] and 15.7% [40]. Four of these studies had sufficient one-stage and two-stage revisions to compare reinfection outcomes.

Elson reported almost three times the rate of reinfection over a range of 1 to 68 months after one-stage as two-stage revisions [34]. He did not present information on patient characteristics, however, and acknowledged that the difference in infection rates represented "an oversimplification of the many complex factors involved in the analysis of these results" [41] (p. 761).

Ketterl and colleagues reported a threefold increase in the risk of reinfection in patients treated with one-stage revision compared with two-stage revision [35]. Again, no information was provided regarding the characteristics of patients who underwent procedures using the different revision methods. Also, no information was provided on patients who did not receive implantation in a planned two-stage procedure. If these were classified as failed two-stage revisions, the reinfection rate would increase from 10.6% to 26.1%.

In the large, multicentre study comprising 458 patients with surgical revision reported by Vielpeau and Lortat-Jacob, the rate of reinfection in patients who underwent one-stage revision was 11.8% compared with 14.9% in those who underwent two-stage revision [40]. In that study, no information was provided on reinfection within 2 years specifically, and about 18% of patients died or were lost to follow-up.

Sanzen and colleagues reported a 13% reduction in reinfection in patients who received a one-stage compared with a two-stage replacement [39]. Unlike the reporters of the previous studies, these authors described the process by which patients were allocated to one- or two-stage revision; in the earlier years of their study, treatment at one hospital was by one-stage revisions and in two hospitals by two-stage procedures [42].

### Comparison of methods

**Figure 1:**
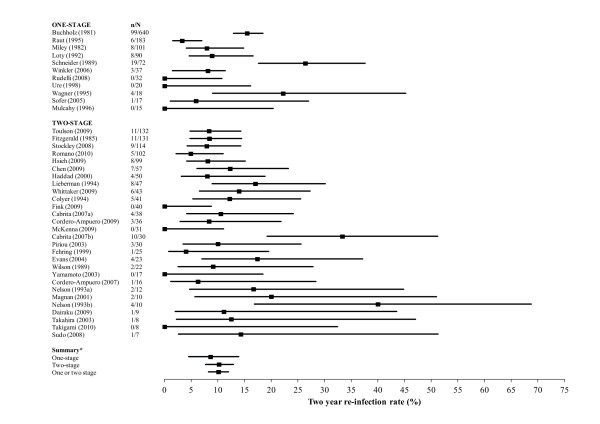
**Proportions of patients with reinfection within 2 years after treatment in studies of one- and two-stage revision in unselected patients**. *N *represents the total number of patients treated surgically, *n *represents the number of patients with reinfection. Cabrita a and b and Nelson a and b refer to randomised intervention and control groups, respectively. *Summary values for one-stage, two-stage and all studies (including studies with unselected patients with one- and two-stage revision; data not shown) were calculated using a random-effects model and the Freeman-Tukey arc-sine transformation to stabilise the variances.

To compare methods, we included all studies with reinfection outcomes, regardless of the number of patients studied. Point estimates and confidence intervals for the proportions of patients with reinfection are shown in Figure 1.

In the 11 studies of exclusively one-stage revision comprising 1,225 patients, the pooled-effect estimate of incidence of reinfection using a random-effects model was 8.6% (95% CI = 4.5 to 13.9). Substantial heterogeneity was evident among the studies (χ^2 ^= 59.06, *df *= 10, *P *= 0.000, *I*^2 ^= 83.1%). In a fixed-effects analysis, the pooled incidence rate was 11.4% (95% CI = 9.7 to 13.3). The discrepancy with the random-effects model was mainly explained by the large study of Buchholz and colleagues. Excluding this study, with its less specific outcome measure of the need for second exchange and other reinfections, the pooled random-effects estimate was 7.7% (95% CI = 3.6 to 13.4) and heterogeneity was reduced slightly (χ^2 ^= 39.61, *df *= 9, *P *= 0.000, *I*^2 ^= 77.3%).

In the 28 studies comprising 1,188 patients treated exclusively with two-stage revision, the pooled random-effects estimate for the incidence of reinfection was 10.2% (95% CI = 7.7 to 12.9). The heterogeneity among studies was moderate (χ^2 ^= 51.73, *df *= 27, *P *= 0.003, *I*^2 ^= 47.8%).

Overall, across the 62 studies comprising 4,197 unselected patients (or hips) treated for prosthesis infection, the reinfection incidence using a random-effects model was 10.1% (95% CI = 8.2 to 12.0) with substantial heterogeneity (χ^2 ^= 209.42, *df *= 61, *P *= 0.000, *I*^2 ^= 70.9%).

We also took the opportunity to compare reinfection rates for all the studies we identified that reported reinfection rates after one- and two-stage revision procedures. This included patients selected by a surgeon for a particular method on the basis of health status and infectious agent. The results of 133 studies comprising 5,556 patients are summarised in Additional file 1, Figure A2. In a random-effects analysis, the rates of reinfection after one- and two-stage revisions were 10.56% (95% CI = 8.12 to 13.31) and 8.71% (95% CI = 7.32 to 10.17), respectively. Overall, across all studies of surgical revision using one- and two-stage methods, the rate of reinfection was 9.19% (95% CI = 7.96 to 10.50). Heterogeneity was moderate (χ^2 ^= 289.67, *df *= 132, *P *= 0.000, *I*^2 ^= 54.4%).

## Discussion

About 10% of surgical revision procedures for infected hip prostheses become reinfected within 2 years. Pooled estimates of reinfection outcomes with wide confidence intervals do not suggest that outcomes are worse after one-stage compared with two-stage revision.

Evidence on the relative effectiveness of one- and two-stage revisions in preventing reinfection of hip prostheses is largely based on interpretation of longitudinal studies. Reviews of longitudinal studies and particularly case series are limited by the possibility of publication bias and other sources of bias. We used systematic review methods to identify studies that described outcomes specifically after one- or two-stage revisions in consecutive and generally unselected patients.

Despite our aim to include only studies of unselected patients undergoing surgical revision, we recognise an earlier phase of selection related to management without further replacement. Different protocols, eligibility criteria and use of treatments with prosthesis retention may affect the representativeness of the study populations we included and may explain the high degree of statistical heterogeneity we observed. As is true of a review of treatment of infected knee prostheses by Jämsen and colleagues, however, we believe that it is important to attempt to include "only papers reporting a pure series of either one-stage or two-stage revision" [43] (p. 72). This is supported by the somewhat different reinfection rates we observed when we included studies that included patients selected for a particular revision method on the basis of infection severity and other aspects of patient health.

In this overview of studies in unselected patients, we specified prevention of reinfection as the key aim of revision surgery. In discussions with our advisory group of patients with joint replacement, patients recognised that clearance of infection is of paramount importance. Achievement of a functional, painless hip is the aim of any revision hip replacement [44], however, and the importance of patient-reported outcomes in assessing joint replacement outcome is widely recognised [45]. In their review, Wolf and colleagues explored patient preferences for one- or two-stage revision in a Markov simulation model [18]. In addition to outcomes reported in longitudinal studies, they used data collected from nonorthopaedic outpatients on trade-offs between impaired health versus full health with shortened life, as well as time with constant severe pain. They concluded that, compared to a two-stage procedure, one-stage revision was associated with greater benefit in terms of quality-adjusted life years.

One- and two-stage methods are perceived to have specific advantages and disadvantages. With a one-stage approach and a single major surgery, recovery is quicker. The advantages of two-stage treatment relate to the opportunity to apply an enhanced antimicrobial strategy between excision and reimplantation surgeries. The results of bacteriological samples obtained at surgery can be used to guide antibiotic treatment, and the period between stages is an opportunity for insertion of antibiotic-impregnated cement beads into the joint space. In two-stage revisions, however, patients experience considerable restriction of movement during the period between implant removal and replacement [4]. Two-stage revision is often considered essential for more virulent infections. Nevertheless, Leung and colleagues reported a reinfection rate of 21% after two-stage revision in patients with resistant infections [46].

To reduce long-term problems resulting from an extended period without an implant, an antibiotic-impregnated cement "spacer" may be used to maintain some function and a correct leg length and to reduce long-term problems associated with disuse. Although spacers improve patient mobility, complications can arise. For example, in 88 spacer implantations performed by Jung and colleagues, there were 15 spacer dislocations (17%), 9 spacer fractures (10%) and an overall complication rate of 58.5% [47].

The economic implications of uncomplicated one- and two-stage revision differ considerably. Although a one-stage procedure may require a prolonged hospital stay to facilitate intravenous antibiotic therapy, the main determinant of cost is the requirement for additional surgery in a two-stage revision. In the United Kingdom, the cost to the NHS of each complicated hip procedure is over £8,000 [48]. Klouche and colleagues estimated that the cost of managing a patient with an infected hip prosthesis is 3.6 times greater than that of a primary total hip replacement and that two-stage revisions cost 1.7 times more than one-stage revisions [14].

Forty-three years of data collection do not conclusively support a specific treatment for prosthetic hip infection. The thoroughness of data collected over extended periods suggests the possibility of individual patient data synthesis with time-to-event analyses [49]. We concur with Matthews and colleagues, however, that large, multicentre, randomised trials are needed to establish optimum management strategies [4], and this is particularly apparent regarding surgical options. It could be argued that appropriate randomised trials are not feasible because of the limited opportunities of an individual surgeon to gain experience in using a particular technique. However, the surgical techniques familiar to surgeons operating with the more widely used two-stage strategy and with aseptic revision (a single-stage revision) include most of the skills required.

The acceptability to patients of methods and their evaluation is of overriding importance. It is notable that only two studies collected data regarding patient-centred outcomes. If reinfection rates are similar between methods, the possibility of a single major surgery, reduced overall hospitalisation and avoidance of a prolonged period without a permanent implant would make a one-stage procedure preferable. Conversely, it could be argued that the long-term, targeted antibiotic regimen associated with contemporary two-stage treatment should not be withheld.

Prosthetic hip infection is sufficiently rare to make a single, definitive randomised trial unlikely. Conducting multiple smaller trials in which patients' experiences and patient-reported outcomes are recorded, together with a systematic overview of infection outcome, may be a more valid approach, and this would also allow exploration of methodological variability and other sources of heterogeneity [50]. Research into the patient experience of prosthetic hip infection and its treatment is urgently required. This will help in the development of studies that identify the best method for treatment of prosthetic joint infection.

## Conclusions

Evidence of the relative effectiveness of one- and two-stage surgical revision in treatment of infected hip prostheses is largely based on interpretation of longitudinal studies. There is no suggestion on the basis of systematic review of published studies that one- or two-stage methods have different reinfection outcomes. Randomised trials are needed to establish optimum management strategies.

## Competing interests

The authors declare that they have no competing interests.

## Authors' contributions

AWB, ADB, RGH, AL and AJS conceived and designed the study. ADB and KTE conducted the systematic review. AJS conducted the statistical analysis. ADB drafted the manuscript with contributions from AWB, KTE, RGH, AJS and AL. All authors read and approved the final version of the manuscript.

## Pre-publication history

The pre-publication history for this paper can be accessed here:

http://www.biomedcentral.com/1741-7015/10/18/prepub

## Supplementary Material

Additional file 1 **Table A1 Meta-analysis of Observational Studies in Epidemiology checklist**. Table A2 Search strategy as applied in MEDLINE. Table A3 Characteristics of all included longitudinal studies regardless of size. Figure A1 Systematic review flow diagram. Figure A2 Proportions of patients with reinfection within 2 years in all studies including patients treated by one- and two-stage revision. Appendix References. Click here for file
