# Supplementary material for: What is the evidence base to guide surgical treatment of infected hip prostheses? systematic review of longitudinal studies in unselected patients
Source: BMC Med. 2012 Feb 16;10:18. doi: 10.1186/1741-7015-10-18 (PMC3364856; doi:10.1186/1741-7015-10-18)
Supplement: Additional file 1 — Table A1 Meta-analysis of Observational Studies in Epidemiology checklist. Table A2 Search strategy as applied in MEDLINE. Table A3 Characteristics of all included longitudinal studies regardless of size. Figure A1 Systematic review flow diagram. Figure A2 Proportions of patients with reinfection within 2 years in all studies including patients treated by one- and two-stage revision. Appendix References. [file 1741-7015-10-18-S1.DOC]

**Additional file**

**Table A**1. MOOSE checklist

| **Reporting of background should include** | |
| --- | --- |
| Problem definition | Background |
| Hypothesis statement | Background |
| Description of study outcome(s) | Re-infection |
| Type of exposure or intervention used | One- and two-stage revision of infected hip prostheses |
| Type of study designs used | Systematic reviews, RCTs, longitudinal studies |
| Study population | Consecutive/ unselected populations |
| **Reporting of search strategy should include** | |
| Qualifications of searchers (eg, librarians and investigators) | Stated in methods |
| Search strategy, including time period included in the synthesis and keywords | Methods and Appendix Table 2 |
| Effort to include all available studies, including contact with authors | We did not contact authors as many studies were over 20 years old  We searched reference lists and citations |
| Databases and registries searched | Methods |
| Search software used, name and version, including special features used (eg, explosion) | Endnote X3/4 |
| Use of hand searching (eg, reference lists of obtained articles) | Methods |
| List of citations located and those excluded, including justification | Flow diagram in Appendix Figure 1. This includes references to all excluded studies and reasons. |
| Method of addressing articles published in languages other than English | No exclusions on basis of language. Authors have some language skills, also colleagues and some use of Google Translate facility |
| Method of handling abstracts and unpublished studies | We included abstracts if clear indication of eligibility |
| Description of any contact with authors | We did not approach authors of studies |
| **Reporting of methods should include** | |
| Description of relevance or appropriateness of studies assembled for assessing the hypothesis to be tested | Results |
| Rationale for the selection and coding of data (eg, sound clinical principles or convenience) | Methods |
| Documentation of how data were classified and coded (eg, multiple raters, blinding, and interrater reliability) | Methods |
| Assessment of confounding (eg, comparability of cases and controls in studies where appropriate) | We included only studies where populations were unselected |
| Assessment of study quality, including blinding of quality assessors; stratification or regression on possible predictors of study results | We included only studies where populations were unselected |
| Assessment of heterogeneity | Insufficient data for heterogeneity analyses relating to treatment. We did report results of contemporary studies and considered the outcome with and without one large study |
| Description of statistical methods (eg, complete description of fixed or random effects models, justification of whether the chosen models account for predictors of study results, dose-response models, or cumulative meta-analysis) in sufficient detail to be replicated | Estimate of overall proportions with outcomes calculated |
| Provision of appropriate tables and graphics | Results summarised in Table 1 and Appendix Table 3 |
| **Reporting of results should include** | |
| Graphic summarizing individual study estimates and overall estimate | Not appropriate |
| Table giving descriptive information for each study included | Table 1 and Appendix Table 3 |
| Results of sensitivity testing (eg, subgroup analysis) | Results (only for date/ exclusion of one large study) |
| Indication of statistical uncertainty of findings | Discussion |
| **Reporting of discussion should include** | |
| Quantitative assessment of bias (eg, publication bias) | Discussion |
| Justification for exclusion (eg, exclusion of non–English-language citations) |  |
| Assessment of quality of included studies | Results and discussion |
| **Reporting of conclusions should include** | |
| Consideration of alternative explanations for observed results | Discussion |
| Generalisation of the conclusions (ie, appropriate for the data presented and within the domain of the literature review) | Discussion |
| Guidelines for future research | Discussion |
| Disclosure of funding source | Acknowledgement |

**Table A2. Search strategy as applied in MEDLINE**

1. prosthesis-related infections/

2. infection/

3. wound infection/

4. surgical wound infection/

5. infect$.mp.

6. exp Arthroplasty, Replacement, Hip/ or exp Hip Prosthesis/ or hip replacement.mp.

7. hip prosthesis.mp. or exp Hip Prosthesis/

8. total hip.tw.

9. 2-stage.tw.

10. 1-stage.tw.

11. two stage.tw.

12. one stage.tw.

13. exchang$.mp.

14. 1 or 2 or 3 or 4 or 5

15. 6 or 7 or 8

16. 9 or 10 or 11 or 12 or 13

17. 14 and 15 and 16

***Table A3. Characteristics of all included longitudinal studies irrespective of size***

| **Study**  **Country**  **Year of study** | **Patients**  **Number of participants**  **Mean age (% men)** | **Exclusive surgical method**  **Other treatment** | **Overall follow up**  **Outcomes**  **Deaths and losses to follow up** | **N (%) re-infection at 2 years**  **Details** | |
| --- | --- | --- | --- | --- | --- |
| **One-stage** |  |  |  |  | |
| Buchholz 1981 [1]  Germany  1968–1977 | Hip arthroplasty for OA (95%), others (5%)  N=640  58.8 years (39.7%) | “patients with deep infection involving arthroplasties of the hip”  Antibiotic-loaded cement | 52 months  Need for further exchange. Re-infection  90 deaths | 99 (15.5%)  Re-operation due to “bad” outcome plus other infection related outcomes | |
| Loty 1992 [2]  France  1980–1988 | THR  N=90  65.7 years (not specified) | "We usually manage infected total hip replacements by a one stage revision."  Systemic antibiotics and antibiotic loaded cement | 47 months, ±29 months  Re-infection, other failure, Merle d'Aubigné-Postel score  4 deaths, 7 lost to follow up | 8 (8.9%)  Infections occurred between 6 and 24 months | |
| Miley 1982 [3]  USA  1969–1979 | Hip surgery for fracture dislocation (47%), OA (36%), others (17%)  N=100 (101 hips)  Men 56.2 years, women 59 years (53%) | "The operating surgeon must be prepared to perform either of these operations (1-stage or Girdlestone), depending on the surgical findings and medical work-up."  Intensive multiple-drug antimicrobial programme | 48.5 months, minimum 32 months  Grading system focusing on quality of life and hip function. Grade IV outcome: drainage, constant pain, further surgery suggested.  11 deaths | 8 hips (7.9%) Grade 4 outcome  No information on re-infection within 2 years | |
| Mulcahy 1996 [4]  Ireland  Dates not specified | THR  N=19 (patients with excision arthroplasty excluded)  64 years (68%) | 4 patients “underwent Girdlestone excision arthroplasty… were considered high risk anaesthetic candidates”  Antibiotic-loaded cement | 4 years 5 months, range 2–7 years  Re-infection, Harris hip score, radiological assessment  1 lost to follow up | 0 (0%)  No re-infections reported | |
| Raut 1995 [5]  Wroblewski 1986[6]  UK  1979–1990 | Cemented primary THR (63%), revision THR (37%)  N=183  64.5 years (48%) | "A prospective study of a one-stage revision..." "A series of infected revisions." "only cemented...treated by the method outlined"  Antibiotic-loaded cement | 93 months, range 24–164 months  Persistent infection, Merle d'Aubigné-Postel score (pain, function and movement), radiology, re-revision, complications.  14 deaths (0 in year 1) | 6 (3.3%)  Re-infection in first 2 years | |
| Rudelli 2008 [7]  Brazil  1989–2000 | Loose THR with severe bone loss  N=32 (no information on 14 patients without severe bone loss)  61.3 years (41%) | "A further 3 patients underwent a Girdlestone procedure."  75% cemented (not antibiotic loaded). Intravenous and oral antibiotics for 6 months. | 103 months, range 63–183 months  Re-infection, Merle d'Aubigné-Postel pain, walk, mobility  3 deaths | 0 (0%)  No re-infection within 2 years from text and survival curve | |
| Schneider 1989 [8]  Switzerland  1973–1988 | Hip implants  N=72 (excluding 13 treated by irrigation alone)  Not specified | “Between 1980 and 1988, out of 42 deep infections”  (data also from 1973 onwards)  Joint irrigation preceding revision. | Follow up interval not specified.  Re-infection (bad outcome) | 19 (26.4%, rate from 1980 16.1%)  No information on re-infection within 2 years | |
| Sofer 2005 [9]  Germany  Not specified | THR (patients with MRSA excluded)  N=17  Not specified | “We performed one-stage revision arthroplasties”  Antibiotic loaded cement | 17.6 months, range 3–33 months  Re-infection, complications, Merle d'Aubigné-Postel score including mobility, radiological outcome, patient satisfaction  1 death | 1 (5.9%)  "early reinfection" "confirmed eradication... in 15 of 16" | |
| Ure 1998 [10]  USA  1979–1990 | THR for OA (65%), others (35%)  N=20  61.4 years (80%) | “a consecutive series of patients who met our inclusion criteria”  Antibiotic loaded cement | 9.9 years, range 3.5 to 17.1 years  Re-infection, UCLA hip rating for pain, walking, function and activity, and complications, radiographs  5 deaths | 0 (0%)  No re-infections reported | |
| Wagner 1995 [11]  Wagner 1997 [12]  Germany  1991–1993 | THR  N=18 (a further 16 were treated without replacement of prostheses or by removal of prosthesis with no replacement due to poor local and general conditions)  64.6 years | “Im zeitraum von 1991 wurden bei 34 patienten infizierte hüft totalendoprothesen operiert und zusätzlich lokal antiseptisch behandelt”  Topical antiseptic hexamethylenbiguanide | Re-infection, radiological studies | 4 (22.2%)  Re-infection occurred between 12 and 47 months | |
| Winkler 2008 [13]  Austria  1998–2004 | THR  N=37  68.5 years(46%) | “Between 1998 and 2004 we treated 37 patients with an infected THR.”  Antibiotic-loaded cement | 4.4 years, range 2–8  Re-infection, radiological examination | 3 (8.1%)  Time of re-infection described | |
| **Two-stage** |  |  |  |  | |
| Cabrita 2007 RCT no spacer [14]  Brazil  1996–2003 | Trial overall THR for trauma (32%), OA (27%), RA (12%), osteonecrosis (9%)  N=30  54.6 years (58%) | “patients diagnosed with chronic infected hip arthroplasties and treated in our Institution”  No spacer | 4 years, range 2–8.5 years  Re-infection, complications  3 deaths | 10 (33.3%)  Infections after 1st and 2nd stage | |
| Cabrita 2007 RCT spacer [14]  Brazil  1996–2003 | Trial overall THR for trauma (32%), OA (27%), RA (12%), osteonecrosis (9%)  N=38  54.6 years (58%) | “patients diagnosed with chronic infected hip arthroplasties and treated in our Institution”  Vancomycin loaded spacer | 4 years, range 2–8.5 years  Re-infection, complications  2 deaths | 4 (10.5%)  Infections after 1st and 2nd stage | |
| Chen 2009 [15]  Taiwan  1993–2005 | THR (excluding patients with proximal femoral allograft reconstruction)  N=57  51.5 years (72%) | “according to the protocol for short term parenteral antibiotics therapy at this institution”  Interim antibiotic-impregnated cement beads | 67.2 months  Re-infection, Harris hip scores, radiological examination  5 deaths and 5 lost to follow up | 7 (12.3%)  Re-infection in 7 patients after 1^st^ stage described in text | |
| Colyer 1994 [16]  USA  Not specified | Hip implants  N=41  57 years (44%) | “consecutive patients were documented  to have infections about hip implants and  treatment was planned with the above protocol”  Attempt to limit implantation interval to 1 month | 3 years, range 12–88 months  Re-infection, radiological evaluation  5 deaths | 5 (12.2%)  Re-infection within 2–years described in text | |
| Cordero-Ampuero 200 9[17]  Spain  1997–2007 | THR  N=36 (12 further patients refused treatments)  71.8 years (36%) | “From January 1997 to January 2007, we treated 36 patients diagnosed with late hip arthroplasty infection”  Oral antibiotics between stages | 4.4 years, range 1–12 years  Re-infection, Harris hip score  4 deaths | 3 (8.3%)  Re-infection after 1st stage which precluded 2nd stage surgery | |
| Cordero-Ampuero 2007 [18]  Spain  1996–2003 | THR  N=16  72 years (25%) | “Consecutive patients were diagnosed with late arthroplasty infection”  Oral intracellularly-effective antibiotics between stages | 4 years, range 2–9 years  Re-infection, Harris hip score, radiological studies | 1 (6.3%)  Re-infection within 2-years described in text | |
| Dairaku 2009 [19]  Japan  2002– | THR after OA (100%)  N=9 (10 hips)  65 years (22%) | “We had used antibiotics-impregnated cement beads until 2002. Since then, an antibiotics impregnated cement spacer has been used”  Antibiotic impregnated cement spacer | 18 months, range 6–68 months  Re-infection, walking, range of hip motion, change of leg length | 1 (11.1%)  Re-infection observed at 1 year | |
| Evans 2004 [20]  USA  1995–2002 | THR  N=23 hips  65 years (55%) | “consecutive total hip and knee periprosthetic infections”  Antibiotic cement components or antibiotic cement-coated components | 2 years minimum  Re-infection, dislocation, transfer, hip flexion | 4 (17.4%)  Days to re-infection shown for individual patients | |
| Fehring 1999 [21]  USA  Not specified | THR  N=25  Not specified | 25 patients managed with cementless stems out of 29 patients with infected arthroplasties  Cementless fixation. Tobramycin-impregnated beads used in some | 41 months, range 24–98 months  Re-infection, Harris hip score  1 lost to follow up | 1 (4%)  Authors describe one patient with re-infected prosthesis at 2 years | |
| Fink 2009 [22]  Germany  2002–2006 | Hip prosthesis for OA (89% of patients followed up), others (11%)  N=40 (excluding 4 patients with false positive preoperative aspirate)  69 years (44%) | “we changed our protocol for treating periprosthetic late infections at the end of 2002 from a two-stage cemented revision to a two-stage cementless revision that involved a standardized procedure."  Spacer. Mostly cementless replacement | 35 months, range 24–60 months  Re-infection, Harris hip score, radiographic outcomes  1 death | 0 (0%)  No evidence of re-infection | |
| Fitzgerald 1985 [23]  USA  1969–1979 | Hip implant  N=131  61 years (50%) | “delayed reconstruction in 131 patients who had an infection after a previous total hip arthroplasty.”  Cemented reconstruction with no added antibiotic | 49 months, range 2–9 years  Re-infection | All hip implants  11 (8.4%)  Re-infection up to 429 days | |
| McDonald 1989 [24]  1969–1985 | Specifically THR for OA (69%), fracture (13%), other (18%)  N=81 (including additional 13 patients)  60.0 years (53%) |  | 5.5 years, range 2.0–13.6 years  Re-infection, complications | THR only  6 (7.4%) estimated from survival curve | |
| Haddad 2000 [25]  UK  1988–1992 | THR for OA (72%), other (28%)  N=50  60 years 46% | “consecutive patients all of whom were referred with an infected total hip replacement and treated using a standardised protocol”  Antibiotic loaded beads and cement ball. Uncemented | 5.8 years, 2–8.7 years  Re-infection, Harris hip score, radiological outcome, complications  2 deaths | 4 (8%)  No information on re-infection within 2 years | |
| Hsieh 2009 [26]  Taiwan  2002–2005 | Prosthetic hip  N=99  61 years (61%) | "use of an ALCS in SEA for PHI has been a routine practice in our institution"  Antibiotic-loaded cement spacer | 43 months, range, 24–60 months  Re-infection, Merle d'Aubigné-Postel score (only in comparison of antibiotic strategies), radiographic results  3 deaths, 5 lost to follow up | 8 (8.1%)  Re-infection between stages | |
| Lieberman 1994 [27]  USA  1985–1988 | THR for OA (70%), RA (11%), others (19%)  N=47 (49 hips)  69 years (47%) | “patients (49 hips) who were treated at The Hospital for Special Surgery for infected THAs”  Gentamicin beads used in 4 patients. Antibiotic-impregnated cement used in 17 | 40 months, range 5–72 months  Re-infection, Harris hip score, complications  4 deaths, 1 loss to follow up | 8 (17.0%)  Intention to treat; includes 5 patients with no 2nd stage due to re-infection | |
| Magnan 2001 [28]  Italy  1996–1999 | THR  N=10  72 years (70%) | “From September 1996 to January 1999 we treated 10 patients …. with an infected total hip arthroplasty.”  Spacer containing antibiotics | 35 months (2–4 years)  Re-infection, radiographs, hip flexion | 2 (20%)  From text. By intention to treat: 2 patients with no 2nd stage due to re-infection | |
| McKenna 2009 [29]  Ireland  2001–2004 | THR  N=30 (31 hips)  63 years (57%) | “a consecutive series of patients presenting with infected hip endo-prostheses for treatment in our institution”  Interim antibiotic eluting cement spacer. Coated uncemented femoral prosthesis | 35 months, 24–60 months  Re-infection, Harris hip score (including pain), range of movement  1 death | 0 (0%)  No evidence of re-infection | |
| Nelson 1993 RCT no antibiotic beads [30]  USA  1985–1990 | THR  N=10  60 years (68%) in RCT overall (including knee patients) | “All patients with infected total joints underwent debridement, resection arthroplasty, and culture.”  Systemic antibiotics | 32 months, range 6 months–5.6 years  Re-infection  4 deaths in RCT overall | 4 (40%)  No information on re-infection within 2 years | |
| Nelson 1993 RCT antibiotic beads [30]  USA  1985–1990 | THR  N=12  60 years (68%) in RCT overall (including knee patients) | “All patients with infected total joints underwent debridement, resection arthroplasty, and culture.”  Gentamicin-impregnated PMMA beads | 32 months, range 6 months–5.6 years  Re-infection  4 deaths in RCT overall | 2 (16.7%)  No information on re-infection within 2 years | |
| Piriou 2003 [31]  France  1987–1997 | THR  N=30  64 years (57%) | “we prospectively followed thirty patients, who had a chronically infected hip arthroplasty treated by the conventional two-stage revision procedure”  Verification of infection eradication with technetium-gallium bone scans. No spacer | 5 years, range 2–13 years  Re-infection, Merle d'Aubigné-Postel score | 3 (10%)  Individual patient re-infection times in text | |
| Romanò 2010 [32]  Italy  2000–2007 | Hip prostheses  N=102  58 years (34%) | “102 consecutive patients underwent two-stage revision of septic hip replacement”  Long stem or short stem preformed antibiotic loaded cement spacers.  Cementless | 48 months  Re-infection, Harris hip score (only in comparison of antibiotic strategies)  3 deaths, 9 lost to follow up | 5 (4.9%)  Re-infection within 3 years (including between stages) | |
| Stockley 2008 [33]  UK  1991–2004 | THR for OA (60%), post traumatic arthritis (18%), others (23%)  N=114  64 years (55%) | “consecutive patients with microbiologically-proven deep chronic infection of the hip were managed by a two-stage exchange procedure.”  Antibiotic loaded cement beads | 74 months, range 2–175 years  Re-infection | 9 (7.9%)  Re-infection within 1 year | |
| Sudo 2008 [34]  Japan  1998–2000 | Hip prosthesis for OA (43%), RA (29%)  N=7  65 years (29%) | “7 consecutive patients who had a deep infection at the site of a hip prosthesis”  Antibiotic impregnated ceramic block  Cementless component, with or without  antibiotic-impregnated ceramic additionally implanted | 5 year, range 2.3–6.1 years  Re-infection, complications, radiographic studies  2 deaths | 1 (14.3%)  Re-infection treated with debridement at 2 years | |
| Takahira 2003 [35]  Japan  1996–2000 | THR including hemiarthroplasty for OA (50%), others (50%)  N=8 (9 hips)  67.1 years (50%) | “technical details and treatment outcomes of our protocol for two-stage revision”  Antibiotic-impregnated cement spacer  Cementless | 35.7 months, range 10–55 months  Re-infection, Japanese Orthopaedic Association score, complications | 1 (12.5%)  Re-infection in 1 patient at 4 months specified in text | |
| Takigami 2010 [36]  Japan  1999–2006 | THR for OA (59.5%)  N=8  65 years (75%) | “consecutive patients with hip prosthesis infection”  Porous ceramic blocks loaded with antibiotic | 49 months, range 24–81 months  Re-infection, Japanese Orthopaedic Association score, radiological study | 0 (0%)  No re-infection up to 24 months | |
| Toulson 2009 [37]  USA  1989–2003 | THR  N=132  54.7 years (59%) in patients “who completed the entire protocol” | “All 132 cases of infected THAs treated at our institution”  Spacer containing antibiotic impregnated  cement used in 67% | 64.8 months, range 24–203 months. 8 patients only followed for average 7.2 months  Re-infection, Harris hip score (mean only)  34 deaths (2 with no infection information), 8 lost to follow up | 11 (8.3%)  3 new infections within 24 months, 4 infection not eradicated, 4 patients who died had infection | |
| Whittaker 2009 [38]  UK  1998–2003 | THR  N=43 (44 hips)  69 years (49%) | “consecutive patients with chronic peri-prosthetic infection of the hip”  Antibiotic-impregnated acrylic cement spacer | Median 49 months, range 25–83 months  Re-infection, Merle d'Aubigné-Postel score, radiological examination, complications  3 deaths | 6 (14.0%)  Persistent or super-infection | |
| Wilson 1989 [39]  USA | THR  N=22 | “patients with deep infection of the hip were reimplanted”  No antibiotic-impregnated cement  Cemented (n=9), uncemented (n=13) | Minimum 3 years  Re-infection | 2 (9.1%)  Recurrent infection | |
| Yamamoto 2003 [40]  Japan  1998–2002 | THR or bipolar prosthesis  N=17  61.8 years (35%) | “17 patients … with a total hip or bipolar prosthesis became infected.”  Antibiotic-impregnated cement spacer | 38 months, range 14–62 months  Re-infection, Harris hip score, radiographs, complications, flexion | 0 (0%)  No information on re-infection within 2 years | |
| **Combination of methods** |  |  |  |  | |
| Antti-Poika 1989 [41]  Finland  1976–1985 | THR for OA (81%), others (19%)  N=33 (a further 3 not treated with surgery)  Median 57 years (39%) | 1-stage (n=5 including 2 with single component revised), 2-stage (n=26) | Median 6 years (range 1–15)  Re-infection, complications  5 deaths, 2 excluded from analysis | Overall 7 (22.6%)  1-stage 2 (40%)  2-stage 5 (19.2%) by intention to treat | |
| Cherney 1983 [42]  USA  1971–1978 | Diverse hip surgery  N=33  57 years (52%) | 1-stage (n=5), 2-stage (n=28) | At least 3 years  Re-infection, pain, walking ability, function | Overall 7 (21.2%)  Unable to separate 1- and 2-stage outcomes | |
| Collin 2002 [43]  France  1992–1999 | THR  N=40  1-stage 63.3 years  2-stage 64.6 years (50%) | 1-stage (n=17), 2-stage (n=15), resection only (n=8) | 26 months, minimum 24 months | Overall 2 (5%)  1-stage 1 (5.9%)  2-stage 1 (6.7%) | |
| Darley 2009 [44]  UK  Not specified | THR  N=25  Not specified | 1-stage (n=6), 2-stage (n=19)  Early switch to oral antibiotics | 12–24 months  Re-infection | Overall 0 (0%)  1-stage 0 (0%)  2-stage 0 (0%) | |
| De Man 2011 [45]  Switzerland  1985–2004 | THR  N=79 hips  70 years (57%) in patients followed up | 1-stage (n=24), 2-stage (n=55)  Some patients had spacer between stages. | Mean 3.8 years (SD 2.2)  Re-infection, Harris hip score including limping and walking, radiographic outcome  7 lost to follow up | Overall 2 (2.5%)  1-stage 1 (4.5%)  2-stage 1 (2.0%) | |
| Elson 1993 [46]  UK  Not specified | THR  N=296 (definite or possible infection)  Not specified | 1-stage (n=235), 2-stage (n=61)  Antibiotic-loaded cement pellets used in 2-stage method | Not specified  Re-infection, mechanical survival, radiological outcome | Overall 36 (12.2%)  1-stage 33 (14.0%)  2-stage 3 (4.9%)  Time of definite or possible re-infection mean 25 months, range 1–68 months | |
| Gao 2008 [47]  China  1999–2005 | THR  N=15  63 years 67%) | 1-stage (n=10), 2-stage (n=5) | 19 months, range 12–37 months  Re-infection, Harris hip score | Overall 0 (0%)  1-stage 0 (0%)  2-stage 0 (0%)  Up to at least 12 months | |
| Garvin 1994 [48]  USA  1983–1986 | THR for OA (45%), fracture or trauma (32.5%), other (22.5%)  N=40  66.9 years (45%) | 1-stage (n=10), 2-stage (n=30)  Gentamicin cement, antibiotic beads | Mean 5.7 years, range 2–10 years  Re-infection, walking, complications, pain, muscle power, range of movement | Overall 2 (5.0%)  1-stage 1 (10.0%)  2-stage 1 (3.3%)  Re-infection within 2 years | |
| Giulieri 2004 [49]  Switzerland  1984–2001 | THR  N=49 (excluding 14 patients with prosthesis retention)  Median 72 years (67%) | 1-stage (n=16), 2-stage (n=31), Girdlestone (n=2)  Choice of surgery and antibiotic therapy based on treatment algorithm. | 28 months, range, 0–156 months  Re-infection  9 deaths in total 63 patients (including those with no surgery) | Overall 5 (10.2%)  1-stage 1 (6.3%)  2-stage 3 (9.7%)  Time of re-infection not specified | |
| Goodman 1988 [50]  USA  1971–1982 | THR with avascular necrosis (53%), OA including post-traumatic (37%), others (21%)  N=19 (21 hips)  Mean 62.5 years (47%) | “inclusive and unselected, consecutive series.” 1-stage (n=2), 2-stage (n=7), resection only (n=6)  Some use of antibiotic containing cement | 4.8 years, range 1.2–11.7 years  Re-infection, Harris hip score | Overall 8 (53.3%)  1-stage 1 (50%)  2-stage 3 (42.9%)  Time of re-infection not specified | |
| Hughes 1979 [51]  USA  1971–1975 | THR with fracture (38%), OA (12%), post-Girdlestone (23%), others (27%)  N=26  62 years (38%) | 1-stage (n=13), 2-stage (n=13) | 51 months, 32–83 months  Re-infection, HSS pain, walking, motion, muscle power and function, radiological review | Overall 2 (7.7%)  1-stage 1 (7.7%)  2-stage 1 (7.7%)  Re-infection within 2 years | |
| Ketterl 1988 [52]  Germany  1976–1986 | THR  N=207  69 years (42%) | 1-stage (n=21), 2-stage (161), no re-implantation (n=25)  Gentamicin cement | 32 months  Re-infection, function | Overall 24 (11.6%)  1-stage 7 (33.3%)  2-stage 17 (10.6%)  Time of re-infection unclear | |
| Ladero Morales 1999 [53]  Spain  1985–1995 | THR  N=62 (further 12 with non-surgical treatment)  74 years (53%) | 1-stage (n=2), 2-stage (n=37), resection only (n=23)  Some treated after introduction of peri-operative antibiotic protocol | 4.8 years  Re-infection, Merle d'Aubigné-Postel clinical and functional outcome (pain, mobility, gait) | Overall 3 (4.8%)  1-stage 0 (0%)  2-stage 3 (8.1%)  Time of re-infection unclear | |
| Lecuire 1999 [54]  France  1982–1997 | THR  N=57  70.6 years | 1-stage (n=16), 2-stage (n=41)  Uncemented | 6.6 years  Re-infection, PMA scale, Harris hip score | Overall 2 (3.5%)  1-stage 1 (6.3%)  2-stage 1 (2.4%)  Time of re-infection unclear | |
| Oussedik 2010 [55]  UK  1999–2002 | THR  N=50  65 years (42%) | 1-stage (n=11), 2-stage (n=39)  Antibiotic loaded spacer used in 2-stage. 1-stage used gentamicin loaded cement | 6.8 years, range 5.5 to 8.8 years  Re-infection, Harris hip score, VAS satisfaction | Overall 2 (4.0%)  1-stage 0 (0%)  2-stage 2 (5.1%)  No information on re-infection within 2 years | |
| Ritter 2010 [56]  USA  1969–2004 | THR  N=33 (35 hips)  66.2 years (52%) | 1-stage (n=5), 2-stage (n=17), resection only (n=8), no data on 3 deaths and 2 losses to follow up | 6.9 years, range 0.1–23.3 years)  Re-infection  3 deaths and 2 losses to follow up within 1 year of final treatment | Overall 12 hips (34.3%)  1-stage 0 (0%)  2-stage 5 (29.4%)  No information on re-infection within 2 years |  |
| Salvati 1982 [57]  USA  1971–1975 | Endoprostheses (46%), THR (19%), previous Girdlestone (19%), other (12%)  N=26  62 years (38%) | 1 stage (n=13), 2-stage (n=13) | Minimum 32 months  Re-infection | Overall 4 (15.4%)  Unable to separate 1- and 2-stage outcomes. Time of re-infection unclear (early follow up considered) | |
| Sanzen 1988 [58]  Carlsson 1978 [59]  Sweden  1974–1981 | THR for OA (74%), congenital dislocation (8%), fracture (8%), others (10%)  N=108 (110 hips)  64 years (53%) | 1-stage (n=78 hips), 2-stage (n=32 hips)  In 44% of 2-stage procedures gentamicin loaded PMMA beads were used. Gentamicin loaded cement | 71 months, range 24–117 months  Re-infection, loosening, function  8 deaths within 24 months | Overall 22 (20.0%)  1-stage 17 (21.8%)  2-stage 8 (25.0%)  At least 22/25 re-infections in first year | |
| Schafroth 1999 [60]  Switzerland  1984–1999 | THR  N=38 (includes some patients with no removal of prosthesis)  Not specified | “38 patients who were treated in our clinic because of infected total hip arthroplasties.” | 4.1 years, range 0.1–11 years  Re-infection  2 deaths | Overall 1 (2.6%)  Unable to separate 1- and 2-stage outcomes | |
| Vielpeau 2002 [61]  France  Up to December 1998 | THR  N=458 (including acetabular or femoral revision only, excluding methods with retention of components)  No age or sex details | 1-stage (n=127), 2-stage (n=222), resection (n=81)  Antibiotic cement (n=249), no antibiotic cement (n=100) in 1- or 2-stage | Median 3 years. 81.5% followed for minimum 2 years  Re-infection, complications | Overall 72 (15.7%)  1-stage 15 (11.8%)  2-stage 33 (14.9%) | |
| Wang 2005 [62]  China  1975–2004 | THR  N=35 (excluding 8 with prosthesis retention)  54 years (42%) | 1-stage (n=7), 2-stage (n=15), resection only (n=13) | 3.2 years, range 5 months–16 years  Re-infection, Harris hip score | Overall 0 (0%)  1-stage 0 (0%)  2-stage 0 (0%)  Up to at least 5 months | |
| Weber 2000 [63]  Switzerland  1990–1994 | THR  N=23 (excluding 5 patients with no removal of prosthesis)  73 years (43%) | 1-stage (n=2), 2-stage (n=14), resection only (n=7) | 46 months, range 7–94 months  Re-infection, function, pain, radiological evaluation  6 deaths | Overall 3 (10.7%)  1-stage 0 (0%)  2-stage 0 (0%)  Time of re-infection unclear | |
| Wilson 1974 [64]  Salvati 1982 [57]  USA  1968–1971 | THR (2 patients with no previous implant)  N=19  63 years (21%) | 1-stage (n=14), 2-stage (n=5) | 3 years, range 2–5 years  HSS pain, walking, motion and function, | Overall 2 (10.5%)  1-stage 2 (14.3%)  2-stage 0 (0%)  Re-infection within 2 years | |

**Figure A1. Systematic review flow diagram**

**Screening**

**Included**

**Eligibility**

**Identification**

Records screened

**523**

Records excluded on basis of title and abstract

**370**

Full text articles assessed for eligibility

**167**

**Relevant articles 66**

**Studies included in review**

1-stage only 11 (13 articles) [A1–A13]

2-stage only 28 (27 articles) [A14–40]

Combination 23 (24 articles) [A41–64]

Reviews 2 [A65,A66]

**Exclusions 102**

Selected one-stage 6 [A67–72]

Selected two-stage 33 [A73–105]

Selected specific infection 8 [A106–113]

Selected late infection only 1 [A114]

Selected massive bone loss 5 [A115–119]

Selected component 2 [A120,A121]

Protocol with repeated revisions 1 [A122]

Follow up of available cases 2 [A123,A124]

Combined hip and knee data 7 [A125–131]

Additional publication 6 [A132–137]

No follow up to 2 years 11 [A138–148]

Not treatment of infection 2 [A149,A150]

Resection (Girdlestone) only 1 [A151]

No infection outcome 4 [A152–155]

Not revision specifically 1 [A156]

Specific implant 1 [A157]

No surgical intervention 1 [A158]

No follow up details <2 years 3 [A159–161]

Infected knee revision 1 [A162]

Not infected prosthesis 1 [A163]

Revision of failed treatment 2 [A164,A165]

Follow up of 2^nd^ stage only 1 [A166]

Unable to acquire article 2 [A167,A168]

Extra records identified from reference lists/ citations

**27**

Records identified through database search to March 2011

**496**

**Figure A2. Proportions of patients with re-infection within 2 years in all studies including patients treated by one- and two-stage revision**

N is total number of patients treated surgically, n is number of patients with re-infection.

Cabrita a and b and Nelson a and b refer to randomised intervention and control groups respectively

*Summary values for one-stage, two-stage and all studies were calculated using a random effects model and the Freeman Tukey arcsin transformation to stabilise the variances.

**Appendix References**

A1. Buchholz H, Elson R, Engelbrecht E, Lodenkamper H, Rottger J, Siegel A: **Management of deep infection of total hip replacement**. *J Bone Joint Surg* 1981, **63-B**(3):342-353.

A2. Loty B, Postel M, Evrard J, Matron P, Courpied JP, Kerboull M, Tomeno B: **One stage revision of infected total hip replacements with replacement of bone loss by allografts. Study of 90 cases of which 46 used bone allografts**. *Int Orthop* 1992, **16**(4):330-338.

A3. Miley GB, Scheller AD, Turner RH: **Medical and surgical treatment of the septic hip with one-stage revision arthroplasty**. *Clin Orthop Relat Res* 1982, **170**(76-82).

A4. Mulcahy DM, O'Byrne JM, Fenelon GE: **One stage surgical management of deep infection of total hip arthroplasty**. *Irish J Med Sci* 1996, **165**(1):17-19.

A5. Raut VV, Siney PD, Wroblewski BM: **One-stage revision of total hip arthroplasty for deep infection. Long-term followup**. *Clin Orthop Relat Res* 1995, **321**:202-207.

A6. Wroblewski BM: **One-stage revision of infected cemented total hip arthroplasty**. *Clin Orthop Relat Res* 1986, **211**:103-107.

A7. Rudelli S, Uip D, Honda E, Lima AL: **One-stage revision of infected total hip arthroplasty with bone graft**. *J Arthroplasty* 2008, **23**(8):1165-1177.

A8. Schneider R: **The infected total prosthesis**. *Orthopade* 1989, **18**(6):527-532.

A9. Sofer D, Regenbrecht B, Pfeil J: **Early results of one-stage septic revision arthroplasties with antibiotic-laden cement. A clinical and statistical analysis**. *Orthopade* 2005, **34**(6):592-602.

A10. Ure KJ, Amstutz HC, S N, Schmalzried TP: **Direct-exchange arthroplasty for the treatment of infection after total hip replacement. An average ten-year follow-up**. *J Bone Joint Surg* 1998, **80-A**(7):961-968.

A11. Wagner M: **Local antisepsis in revision surgery of infected total hip prostheses**. *Orthopade* 1995, **24**(4):319-325.

A12. Wagner M, Willenegger H: **Local antisepsis in revision of infected total hip replacement**. *Acta Chir Austriaca* 1997, **29** (Suppl 133):64-68.

A13. Winkler H, Stoiber A, Kaudela K, Winter F, Menschik F: **One stage uncemented revision of infected total hip replacement using cancellous allograft bone impregnated with antibiotics**. *J Bone Joint Surg* 2008, **90-B**(12):1580-1584.

A14. Cabrita HB, Croci AT, Camargo OP, Lima AL: **Prospective study of the treatment of infected hip arthroplasties with or without the use of an antibiotic-loaded cement spacer**. *Clinics* 2007, **62**(2):99-108.

A15. Chen WS, Fu TH, Wang JW: **Two-stage reimplantation of infected hip arthroplasties**. *Chang Gung Medical Journal* 2009, **32**(2):188-197.

A16. Colyer RA, Capello WN: **Surgical treatment of the infected hip implant. Two-stage reimplantation with a one-month interval**. *Clin Orthop Relat Res* 1994, **298**:75-79.

A17. Cordero-Ampuero J, Esteban J, Garcia-Cimbrelo E: **Oral antibiotics are effective for highly resistant hip arthroplasty infections**. *Clin Orthop Relat Res* 2009, **467**(9):2335-2342.

A18. Cordero-Ampuero J, Esteban J, Garcia-Cimbrelo E, Munuera L, Escobar R: **Low relapse with oral antibiotics and two-stage exchange for late arthroplasty infections in 40 patients after 2-9 years**. *Acta Orthop* 2007, **78**(4):511-519.

A19. Dairaku K, Takagi M, Kawaji H, Sasaki K, Ishii M, Ogino T: **Antibiotics-impregnated cement spacers in the first step of two-stage revision for infected totally replaced hip joints: report of ten trial cases**. *J Orthop Sci* 2009, **14**(6):704-710.

A20. Evans RP: **Successful treatment of total hip and knee infection with articulating antibiotic components: a modified treatment method**. *Clin Orthop Relat Res* 2004, **427**:37-46.

A21. Fehring TK, Calton TF, Griffin WL: **Cementless fixation in 2-stage reimplantation for periprosthetic sepsis**. *J Arthroplasty* 1999, **14**(2):175-181.

A22. Fink B, Grossmann A, Fuerst M, Schafer P, Frommelt L: **Two-stage cementless revision of infected hip endoprostheses**. *Clin Orthop Relat Res* 2009, **467**(7):1848-1858.

A23. Fitzgerald RH, Jones DR: **Hip implant infection: Treatment with resection arthroplasty and late total hip arthroplasty**. *Am J Med* 1985, **78**(6):225-228.

A24. McDonald DJ, Fitzgerald RH, Ilstrup DM: **Two-stage reconstruction of a total hip arthroplasty because of infection**. *J Bone Joint Surg* 1989, **71-A**(6):828-834.

A25. Haddad FS, Muirhead-Allwood SK, Manktelow AR, Bacarese-Hamilton I: **Two-stage uncemented revision hip arthroplasty for infection**. *J Bone Joint Surg* 2000, **82-B**(5):689-694.

A26. Hsieh PH, Huang KC, Lee PC, Lee MS: **Two-stage revision of infected hip arthroplasty using an antibiotic-loaded spacer: Retrospective comparison between short-term and prolonged antibiotic therapy**. *J Antimicrob Chemother* 2009, **64**(2):392-397.

A27. Lieberman JR, Callaway GH, Salvati EA, Pellicci PM, Brause BD: **Treatment of the infected total hip arthroplasty with a two-stage reimplantation protocol**. *Clin Orthop Relat Res* 1994, **301**:205-212.

A28. Magnan B, Regis D, Biscaglia R, Bartolozzi P: **Preformed acrylic bone cement spacer loaded with antibiotics: use of two-stage procedure in 10 patients because of infected hips after total replacement**. *Acta Orthop Scand* 2001, **72**(6):591-594.

A29. McKenna PB, O'Shea K, Masterson EL: **Two-stage revision of infected hip arthroplasty using a shortened post-operative course of antibiotics**. *Arch Orthop Trauma Surg* 2009, **129**(4):489-494.

A30. Nelson CL, Evans RP, Blaha JD, Calhoun J, Henry SL, Patzakis MJ: **A comparison of gentamicin-impregnated polymethylmethacrylate bead implantation to conventional parenteral antibiotic therapy in infected total hip and knee arthroplasty**. *Clin Orthop Relat Res* 1993, **295**:96-101.

A31. Piriou P, de Loynes B, Garreau de Loubresse C, Judet T: **Use of combined gallium-technetium scintigraphy to determine the interval before second-stage prosthetic reimplantation in hip arthroplasty infection: a consecutive series of 30 cases**. *Rev Chir Orthop Reparatrice Appar Mot* 2003, **89**(4):287-296.

A32. Romanò CL, Romanò D, Logoluso N, Meani E: **Long-stem versus short-stem preformed antibiotic-loaded cement spacers for two-stage revision of infected total hip arthroplasty**. *HIP Int* 2010, **20**(1):26-33.

A33. Stockley I, Mockford BJ, Hoad-Reddick A, Norman P: **The use of two-stage exchange arthroplasty with depot antibiotics in the absence of long-term antibiotic therapy in infected total hip replacement**. *J Bone Joint Surg* 2008, **90-B**(2):145-148.

A34. Sudo A, Hasegawa M, Fukuda A, Uchida A: **Treatment of infected hip arthroplasty with antibiotic-impregnated calcium hydroxyapatite**. *J Arthroplasty* 2008, **23**(1):145-150.

A35. Takahira N, Itoman M, Higashi K, Uchiyama K, Miyabe M, Naruse K: **Treatment outcome of two-stage revision total hip arthroplasty for infected hip arthroplasty using antibiotic-impregnated cement spacer**. *J Orthop Sci* 2003, **8**(1):26-31.

A36. Takigami I, Ito Y, Ishimaru D, Ogawa H, Mori N, Shimizu T, Terabayashi N, Shimizu K: **Two-stage revision surgery for hip prosthesis infection using antibiotic-loaded porous hydroxyapatite blocks**. *Arch Orthop Trauma Surg* 2010, **130**(10):1221-1226.

A37. Toulson C, Walcott-Sapp S, Hur J, Salvati E, Bostrom M, Brause B, Westrich GH: **Treatment of infected total hip arthroplasty with a 2-stage reimplantation protocol: update on "our institution's" experience from 1989 to 2003**. *J Arthroplasty* 2009, **24**(7):1051-1060.

A38. Whittaker JP, Warren RE, Jones RS, Gregson PA: **Is prolonged systemic antibiotic treatment essential in two-stage revision hip replacement for chronic Gram-positive infection?** *J Bone Joint Surg* 2009, **91-B**(1):44-51.

A39. Wilson MG, Dorr LD: **Reimplantation of infected total hip arthroplasties in the absence of antibiotic cement**. *J Arthroplasty* 1989, **4**(3):263-269.

A40. Yamamoto K, Miyagawa N, Masaoka T, Katori Y, Shishido T, Imakiire A: **Clinical effectiveness of antibiotic-impregnated cement spacers for the treatment of infected implants of the hip joint**. *J Orthop Sci* 2003, **8**(6):823-828.

A41. Antti-Poika I, Santavirta S, Konttinen YT, Honkanen V: **Outcome of the infected hip arthroplasty. A retrospective study of 36 patients**. *Acta Orthop Scand* 1989, **60**(6):670-675.

A42. Cherney DL, Amstutz HC: **Total hip replacement in the previously septic hip**. *J Bone Joint Surg* 1983, **65-A**(9):1256-1265.

A43. Collin P, Siret P, Lahogue J-F, Lambotte J-C, Thomazeau H, Langlais F: **Infected hip prosthesis. One- or two-stage replacement? Comparison of 2 series**. *Ann Orthop Ouest* 2002, **34**:129-134.

A44. Darley E, Bannister G, Blom A, MacGowan AP, Jacobson S, Alfouzan W: **Early intravenous to oral antibiotic switch therapy is effective in the treatment of infected total hip replacement**. *Clin Microbiol Infect* 2009, **15**(Suppl S4):S139.

A45. De Man FH, Sendi P, Zimmerli W, Maurer TB, Ochsner PE, Ilchmann T: **Infectiological, functional, and radiographic outcome after revision for prosthetic hip infection according to a strict algorithm**. *Acta Orthop* 2011, **82**(1):27-34.

A46. Elson RA: **One-stage exchange in the treatment of the infected total hip arthroplasty**. *Sem Arthroplasty* 1994, **5**:137-141.

A47. Gao H, Lv H: **One-stage revision operations for infection after hip arthroplasty**. *Chinese Journal of Reparative & Reconstructive Surgery* 2008, **22**(1):5-8.

A48. Garvin KL, Evans BG, Salvati EA, Brause BD: **Palacos gentamicin for the treatment of deep periprosthetic hip infections**. *Clin Orthop Relat Res* 1994, **298**:97-105.

A49. Giulieri SG, Graber P, Ochsner PE, Zimmerli W: **Management of infection associated with total hip arthroplasty according to a treatment algorithm**. *Infection* 2004, **32**(4):222-228.

A50. Goodman SB, Schurman DJ: **Outcome of infected total hip arthroplasty. An inclusive, consecutive series**. *J Arthroplasty* 1988, **3**(2):97-102.

A51. Hughes PW, Salvati EA, Wilson PD, Blumenfeld EL: **Treatment of Subacute Sepsis of the Hip by Antibiotics and Joint Replacement Criteria For Diagnosis With Evaluation of Twenty-Six Cases**. *Clin Orthop Relat Res* 1979, **141**:143-157.

A52. Ketterl R, Henly MB, Stübinger B, Beckurts T, Claudi B: **Analysis of three operative techniques for infected total hip replacements**. *Orthop Trans* 1988, **12**:715.

A53. Ladero Morales F, Fernandez Gonzalez J, Blanco Ortiz F, Martinez Martin J, Garcia Araujo C: **Treatment of infected hip arthroplasty. Retrospective study**. *Revista de Ortopedia y Traumatologia* 1999, **43**(2):84-92.

A54. Lecuire F, Collodel M, Basso M, Rubini J, Gontier D, Carrere J: **Revision of infected total hip prostheses by ablation reimplantation of an uncemented prosthesis. 57 case reports**. *Rev Chir Orthop Reparatrice Appar Mot* 1999, **85**(4):337-348.

A55. Oussedik SI, Dodd MB, Haddad FS: **Outcomes of revision total hip replacement for infection after grading according to a standard protocol**. *J Bone Joint Surg* 2010, **92-B**(9):1222-1226.

A56. Ritter MA, Farris A: **Outcome of infected total joint replacement**. *Orthopedics* 2010, **33**(3).

A57. Salvati EA, Chekofsky KM, Brause BD, Wilson PD: **Reimplantation in infection: a 12-year experience**. *Clin Orthop Relat Res* 1982, **170**:62-75.

A58. Sanzen L, Carlsson A, Josefsson G, Lindberg LT: **Revision operations on infected total hip arthroplasties**. *Clin Orthop Relat Res* 1988, **229**:165-172.

A59. Carlsson A, Josefsson G, Lindberg L: **Revision with gentamicin-impregnated cement for deep infections in total hip arthroplasties**. *J Bone Joint Surg* 1978, **60-A**(8):1059-1064.

A60. Schafroth M, Zimmerli W, Ochsner PE: **The infected artificial hip joint: possibilities, follow-up and results of treatment**. *Praxis* 1999, **88**(51-52):2101-2105.

A61. Vielpeau C, Lortat-Jacob A: **Management of the infected hip prostheses**. *Rev Chir Orthop Reparatrice Appar Mot* 2002, **88**(Suppl 1):159-216.

A62. Wang Y, Hao L, Zhou Y, Li J, Wang J, Tang P, Huang P: **Clinical experience of treating infection after total hip arthroplasty**. *Chinese Journal of Surgery* 2005, **43**(20):1313-1316.

A63. Weber E, Cometta A, Blanc CH, Leyvraz PF: **Review of infected total arthroplasties of the hip and knee-apropos of 28 cases**. *Swiss Surgery* 2000, **6**(6):335-342.

A64. Wilson PD, Aglietti P, Salvati EA: **Subacute sepsis of the hip treated by antibiotics and cemented prosthesis**. *J Bone Joint Surg* 1974, **56-A**(5):879-898.

A65. Gallo J, Smizansky M, Radova L, Potomkova J: **Comparison of therapeutic strategies for hip and knee prosthetic joint infection**. *Acta Chir Orthop Traumatol Cech* 2009, **76**(4):302-309.

A66. Wolf CF, Gu NY, Doctor JN, Manner PA, Leopold SS: **Comparison of one and two-stage revision of total hip arthroplasty complicated by infection: a Markov expected-utility decision analysis**. *J Bone Joint Surg* 2011, **93-A**(7):631-639.

A67. Callaghan JJ, Katz RP, Johnston RC: **One-stage revision surgery of the infected hip. A minimum 10-year followup study**. *Clin Orthop Relat Res* 1999, **369**:139-143.

A68. Garcia S, Soriano A, Esteban P, Almela M, Gallart X, Mensa J: **Usefulness of adding antibiotic to cement in one stage exchange of chronic infection in total hip arthroplasty**. *Medicina Clinica* 2005, **125**(4):138-139.

A69. Katz RP, Callaghan JJ, Johnston RC: **A minimum ten year follow-up study of one stage reimplantation of the infected total hip**. *Orthop Trans* 1994, **18**:993.

A70. Schneider R: **The infected total hip replacement prosthesis**. *Helvetica Chirurgica Acta* 1978, **45**(4-5):553-566.

A71. Wu CC, Chen WJ: **One-stage revision surgery to treat hip infected nonunion after stabilization with a sliding compression screw**. *Arch Orthop Trauma Surg* 2003, **123**(8):383-387.

A72. Yoo JJ, Kwon YS, Koo KH, Yoon KS, Kim YM, Kim HJ: **One-stage cementless revision arthroplasty for infected hip replacements**. *Int Orthop* 2009, **33**(5):1195-1201.

A73. Alexeeff M, Mahomed N, Morsi E, Garbuz D, Gross A: **Structural allograft in two-stage revisions for failed septic hip arthroplasty**. *J Bone Joint Surg* 1996, **78-B**(2):213-216.

A74. Ammon P, Stockley I: **Allograft bone in two-stage revision of the hip for infection. Is it safe?** *J Bone Joint Surg* 2004, **86-B**(7):962-965.

A75. Biring GS, Kostamo T, Garbuz DS, Masri BA, Duncan CP: **Two-stage revision arthroplasty of the hip for infection using an interim articulated Prostalac hip spacer: a 10- to 15-year follow-up study**. *J Bone Joint Surg* 2009, **91-B**(11):1431-1437.

A76. Buttaro MA, Pusso R, Piccaluga F: **Vancomycin-supplemented impacted bone allografts in infected hip arthroplasty. Two-stage revision results**. *J Bone Joint Surg* 2005, **87-B**(3):314-319.

A77. D'Angelo F, Negri L, Zatti G, Grassi FA: **Two-stage revision surgery to treat an infected hip implant. A comparison between a custom-made spacer and a pre-formed one**. *Chirurgia Degli Organi di Movimento* 2005, **90**(3):271-279.

A78. Disch AC, Matziolis G, Perka C: **Two-stage operative strategy without local antibiotic treatment for infected hip arthroplasty: clinical and radiological outcome**. *Arch Orthop Trauma Surg* 2007, **127**(8):691-697.

A79. Ejerhed L, Ahnfelt L: **Results of two-stage procedure in revision THR for infections**. *Acta Orthop Scand* 1991, **62** (Suppl 246):10.

A80. English H, Timperley AJ, Dunlop D, Gie G: **Impaction grafting of the femur in two-stage revision for infected total hip replacement**. *J Bone Joint Surg* 2002, **84-B**(5):700-705.

A81. Estes CS, Beauchamp CP, Clarke HD, Spangehl MJ: **A two-stage retention debridement protocol for acute periprosthetic joint infections**. *Clin Orthop Relat Res* 2010, **468**(8):2029-2038.

A82. Etienne G, Waldman B, Rajadhyaksha AD, Ragland PS, Mont MA: **Use of a functional temporary prosthesis in a two-stage approach to infection at the site of a total hip arthroplasty**. *J Bone Joint Surg* 2003, **85-A** (Suppl 4):94-96.

A83. Hartman CW, Garvin KL: **Dislocation of the hip after reimplantation for infection: an analysis of risk factors**. *Clin Orthop Relat Res* 2006, **447**:24-27.

A84. Hsieh P-H, Chang Y-H, Chen S-H, Ueng SWN, Shih C-H: **High concentration and bioactivity of vancomycin and aztreonam eluted from Simplex cement spacers in two-stage revision of infected hip implants: a study of 46 patients at an average follow-up of 107 days**. *J Orthop Res* 2006, **24**(8):1615-1621.

A85. Ilyas I, Morgan DA: **Massive structural allograft in revision of septic hip arthroplasty**. *Int Orthop* 2001, **24**(6):319-322.

A86. Isiklar ZU, Demirors H, Akpinar S, Tandogan RN, Alparslan M: **Two-stage treatment of chronic staphylococcal orthopaedic implant-related infections using vancomycin impregnated PMMA spacer and rifampin containing antibiotic protocol**. *Bull Hosp Jt Dis* 1999, **58**(2):79-85.

A87. Ivarsson I, Wahlstrom O, Djerf K, Jacobsson SA: **Revision of infected hip replacement. Two-stage procedure with a temporary gentamicin spacer**. *Acta Orthop Scand* 1994, **65**(1):7-8.

A88. Jahoda D, Sosna A, Landor I, Vavrik P, Pokorny D, Hudec T: **Two-stage reimplantation using spacers - the method of choice in treatment of hip joint prosthesis-related infections. Comparison with methods used from 1979 to 1998**. *Acta Chir Orthop Traumatol Cech* 2003, **70**(1):17-24.

A89. Karpas K, Sponer P: **Management of the infected hip arthroplasty by two-stage reimplantation**. *Acta Medica* 2003, **46**(3):113-115.

A90. Kendall RW, Duncan CP, Beauchamp CP: **Bacterial growth on antibiotic-loaded acrylic cement. A prospective in vivo retrieval study**. *J Arthroplasty* 1995, **10**(6):817-822.

A91. Kent M, Rachha R, Sood M: **A technique for the fabrication of a reinforced moulded articulating cement spacer in two-stage revision total hip arthroplasty**. *Int Orthop* 2010, **34**(7):949-953.

A92. Koo KH, Yang JW, Cho SH, Song HR, Park HB, Ha YC, Chang JD, Kim SY, Kim YH: **Impregnation of vancomycin, gentamicin, and cefotaxime in a cement spacer for two-stage cementless reconstruction in infected total hip arthroplasty**. *J Arthroplasty* 2001, **16**(7):882-892.

A93. Lai KA, Shen WJ, Yang CY, Lin RM, Lin CJ, Jou IM: **Two-stage cementless revision THR after infection. 5 recurrences in 40 cases followed 2.5-7 years**. *Acta Orthop Scand* 1996, **67**(4):325-328.

A94. Leunig M, Chosa E, Speck M, Ganz R: **A cement spacer for two-stage revision of infected implants of the hip joint**. *Int Orthop* 1998, **22**(4):209-214.

A95. Levine BR, Della Valle CJ, Hamming M, Sporer SM, Berger RA, Paprosky WG: **Use of the extended trochanteric osteotomy in treating prosthetic hip infection**. *J Arthroplasty* 2009, **24**(1):49-55.

A96. Lim S-J, Park J-C, Moon Y-W, Park Y-S: **Treatment of periprosthetic hip infection caused by resistant microorganisms using 2-stage reimplantation protocol**. *J Arthroplasty* 2009, **24**(8):1264-1269.

A97. Maricevic A, Erceg M, Kljakovic M: **Results of treatment of hip joint prosthesis-related infections**. *Lijecnicki Vjesnik* 1999, **121**(11-12):342-345.

A98. Nestor BJ, Hanssen AD, Ferrergonzalez R, Fitzgerald RH: **The use of porous prostheses in delayed reconstruction of total hip replacements that have failed because of infection**. *J Bone Joint Surg* 1994, **76-A**(3):349-359.

A99. Oussedik SIS, Haddad FS: **The use of linezolid in the treatment of infected total joint arthroplasty**. *J Arthroplasty* 2008, **23**(2):273-278.

A100. Romanò CL, Romanò D, Logoluso N, Meani E: **Septic versus aseptic hip revision: How different?** *J Orthop Trauma* 2010, **11**(3):167-174.

A101. Sanchez-Sotelo J, Berry DJ, Hanssen AD, Cabanela ME: **Midterm to long-term followup of staged reimplantation for infected hip arthroplasty**. *Clin Orthop Relat Res* 2009, **467**(1):219-224.

A102. Thabe H, Schill S: **Two-stage reimplantation with an application spacer and combined with delivery of antibiotics in the management of prosthetic joint infection**. *Operative Orthopadie und Traumatologie* 2007, **19**(1):78-100.

A103. Volin SJ, Hinrichs SH, Garvin KL: **Two-stage reimplantation of total joint infections: a comparison of resistant and non-resistant organisms**. *Clin Orthop Relat Res* 2004, **427**:94-100.

A104. Wei W, Kou B-L, Ju R-S, Lu H-S: **The second stage revision for infected total hip arthroplasty using antibiotic-loaded cement prosthesis**. *Chinese Journal of Surgery* 2007, **45**(4):246-248.

A105. Younger AS, Duncan CP, Masri BA, McGraw RW: **The outcome of two-stage arthroplasty using a custom-made interval spacer to treat the infected hip**. *J Arthroplasty* 1997, **12**(6):615-623.

A106. Hope PG, Kristinsson KG, Norman P, Elson RA: **Deep infection of cemented total hip arthroplasties caused by coagulase-negative staphylococci**. *J Bone Joint Surg* 1989, **71-B**(5):851-855.

A107. Lecuire F, Gontier D, Carrere J, Basso M, Benareau I, Rubini J: **Joint prosthesis infection with Staphyococcus lugdunensis: 7 cases**. *Rev Chir Orthop Reparatrice Appar Mot* 2007, **93**(1):88-92.

A108. Leung F, Richards CJ, Garbuz DS, Masri BA, Duncan CP: **Two-stage total hip arthroplasty: How often does it control methicillin-resistant infection?** *Clin Orthop Relat Res* 2011, **469**(4):1009-1015.

A109. Parvizi J, Azzam K, Ghanem E, Austin MS, Rothman RH: **Periprosthetic infection due to resistant staphylococci: serious problems on the horizon**. *Clin Orthop Relat Res* 2009, **467**(7):1732-1739.

A110. Raut VV, Siney PD, Wroblewski BM: **One-stage revision of infected total hip replacements with discharging sinuses**. *J Bone Joint Surg* 1994, **76-B**(5):721-724.

A111. Raut VV, Orth MS, Orth MC, Siney PD, Wroblewski BM: **One stage revision arthroplasty of the hip for deep gram negative infection**. *Int Orthop* 1996, **20**(1):12-14.

A112. Teterycz D, Ferry T, Lew D, Stern R, Assal M, Hoffmeyer P, Bernard L, Uckay I: **Outcome of orthopedic implant infections due to different staphylococci**. *Int J Infect Dis* 2010, **14**(10):e913-e918.

A113. Zeller V, Lavigne M, Leclerc P, Lhotellier L, Graff W, Ziza JM, Desplaces N, Mamoudy P: **Group B streptococcal prosthetic joint infections: a retrospective study of 30 cases**. *Presse Medicale* 2009, **38**(11):1577-1584.

A114. Tsukayama DT, Estrada R, Gustilo RB: **Infection after total hip arthroplasty. A study of the treatment of one hundred and six infections**. *J Bone Joint Surg* 1996, **78-A**(4):512-523.

A115. Berry DJ, Chandler HP, Reilly DT: **The use of bone allografts in two-stage reconstruction after failure of hip replacements due to infection**. *J Bone Joint Surg* 1991, **73-A**(10):1460-1468.

A116. Hsieh PH, Shih CH, Chang YH, Lee MS, Yang WE, Shih HN: **Treatment of deep infection of the hip associated with massive bone loss: two-stage revision with an antibiotic-loaded interim cement prosthesis followed by reconstruction with allograft**. *J Bone Joint Surg* 2005, **87-B**(6):770-775.

A117. Litt R, Albassir A: **Results of the use of the Muller femoral megaprosthesis. A 2-stage revision for sepsis**. *Acta Orthop Belgica* 1986, **52**(3):383-390.

A118. Michalak KA, Khoo PPC, Yates PJ, Day RE, Wood DJ: **Iontophoresed segmental allografts in revision arthroplasty for infection**. *J Bone Joint Surg* 2006, **88-B**(11):1430-1437.

A119. Nusem I, Morgan DAF: **Structural allografts for bone stock reconstruction in two-stage revision for infected total hip arthroplasty: good outcome in 16 of 18 patients followed for 5-14 years**. *Acta Orthop* 2006, **77**(1):92-97.

A120. Anagnostakos K, Jung J, Kelm J, Schmitt E: **Two-stage treatment protocol for isolated septic acetabular cup loosening**. *HIP Int* 2010, **20**(3):320-326.

A121. Kraay MJ, Goldberg VM, Fitzgerald SJ, Salata MJ: **Cementless two-staged total hip arthroplasty for deep periprosthetic infection**. *Clin Orthop Relat Res* 2005, **441**:243-249.

A122. Walter G, Bühler M, Hoffmann R: **Two-stage procedure to exchange septic total hip arthroplasties with late periprosthetic infection. Early results after implantation of a reverse modular hybrid endoprosthesis**. *Unfallchirurg* 2007, **110**(6):537-546.

A123. Ganse B, Behrens P, Benthien JP: **Two-stage hip revision arthroplasty: The role of the excision arthroplasty**. *Eur J Orthop Surg Traumatol* 2008, **18**(3):223-228.

A124. Hofmann AA, Goldberg TD, Tanner AM, Cook TM: **Ten-year experience using an articulating antibiotic cement hip spacer for the treatment of chronically infected total hip**. *J Arthroplasty* 2005, **20**(7):874-879.

A125. Bejon P, Berendt A, Atkins BL, Green N, Parry H, Masters S, Mclardy-Smith P, Gundle R, Byren I: **Two-stage revision for prosthetic joint infection: predictors of outcome and the role of reimplantation microbiology**. *J Antimicrob Chemother* 2010, **65**(3):569-575.

A126. Berbari EF, Marculescu C, Sia I, Lahr BD, Hanssen AD, Steckelberg JM, Gullerud R, Osmon DR: **Culture-negative prosthetic joint infection**. *Clin Infect Dis* 2007, **45**(9):1113-1119.

A127. Berbari EF, Osmon DR, Duffy MCT, Harmssen RNW, Mandrekar JN, Hanssen AD, Steckelberg JM: **Outcome of prosthetic joint infection in patients with rheumatoid arthritis: the impact of medical and surgical therapy in 200 episodes**. *Clin Infect Dis* 2006, **42**(2):216-223.

A128. Betsch BY, Eggli S, Siebenrock KA, Tauber MG, Muhlemann K: **Treatment of joint prosthesis infection in accordance with current recommendations improves outcome**. *Clin Infect Dis* 2008, **46**(8):1221-1226.

A129. Bohler M, Luschnig J, Knahr K: **Deep implant infections of endoprostheses - Therapy guidelines and results**. *Acta Chir Austriaca* 1997, **29**(SUPPL 133):53-56.

A130. El Helou OC, Berbari EF, Marculescu CE, El Atrouni WI, Razonable RR, Steckelberg JM, Hanssen AD, Osmon DR: **Outcome of enterococcal prosthetic joint infection: is combination systemic therapy superior to monotherapy?** *Clin Infect Dis* 2008, **47**(7):903-909.

A131. Kordelle J, Frommelt L, Kluber D, Seemann K: **Results of one-stage endoprosthesis revision in periprosthetic infection cause by methicillin-resistant Staphylococcus aureus**. *Z Orthop Ihre Grenzgeb* 2000, **138**(3):240-244.

A132. Babst R, Jenny H, Morscher E: **Treatment of infected hip joint arthroplasty. Results of treatment of 62 infected total prosthesis arthroplasties**. *Orthopade* 1989, **18**(6):517-526.

A133. Hsieh PH, Huang KC, Tai CL: **Liquid gentamicin in bone cement spacers: in vivo antibiotic release and systemic safety in two-stage revision of infected hip arthroplasty**. *J Trauma Inj Infect Crit Care* 2009, **66**(3):804-808.

A134. Masri BA, Panagiotopoulos KP, Greidanus NV, Garbuz DS, Duncan CP: **Cementless two-stage exchange arthroplasty for infection after total hip arthroplasty**. *J Arthroplasty* 2007, **22**(1):72-78.

A135. Morscher E, Herzog R, Bapst R: **Hip revision surgery in septic loosening**. *Chirurgia Degli Organi di Movimento* 1994, **79**(4):335-340.

A136. Morscher E, Babst R, Jenny H: **Treatment of infected joint arthroplasty**. *Int Orthop* 1990, **14**(2):161-165.

A137. Winkler H, Kaudela K, Stoiber A, Menschik F: **Bone grafts impregnated with antibiotics as a tool for treating infected implants in orthopedic surgery - One stage revision results**. *Cell and Tissue Banking* 2006, **7 (4)**:319-323.

A138. Bozic KJ, Ries MD: **The impact of infection after total hip arthroplasty on hospital and surgeon resource utilization**. *J Bone Joint Surg* 2005, **87-A**(8):1746-1751.

A139. Della Valle CJ, Bogner E, Desai P, Lonner JH, Adler E, Zuckerman JD, Di Cesare PE: **Analysis of frozen sections of intraoperative specimens obtained at the time of reoperation after hip or knee resection arthroplasty for the treatment of infection**. *J Bone Joint Surg* 1999, **81-A**(5):684-689.

A140. Hsieh P-H, Shih C-H, Chang Y-H, Lee MS, Shih H-N, Yang W-E: **Two-stage revision hip arthroplasty for infection: comparison between the interim use of antibiotic-loaded cement beads and a spacer prosthesis**. *J Bone Joint Surg* 2004, **86-A**(9):1989-1997.

A141. Jahoda D, Sosna A, Landor I, Vavrik P, Pokorny D: **Cannulated articulating spacer: An implant for the treatment of an infected total hip arthroplasty**. *Acta Chir Orthop Traumatol Cech* 2004, **71**(2):73-79.

A142. Kordelle J, Klett R, Stahl U, Sternkopf U, Haas H, Jurgensen I, Schleicher I: **Stage diagnostics for postinfection revision of hip and knee replacement: value of laboratory parameters and antigranulocyte scintigraphy**. *Z Orthop Ihre Grenzgeb* 2003, **141**(5):547-553.

A143. Lecuire F, Rubini J, Basso M, Benareau I: **Traction-mobilization in 2-stage treatment of infected total knee prosthesis. Apropos of 12 cases**. *Revue de Chirurgie Orthopedique et Reparatrice de l Appareil Moteur* 1999, **85**(6):640-645.

A144. Lee G-C, Pagnano MW, Jacofsky DJ, Hanssen AD: **Use of erythropoietin in two-stage reimplantation total hip arthroplasty**. *Clin Orthop Relat Res* 2003, **414**:49-54.

A145. Lenoble E, Goutallier D: **Replacement of infected total hip prosthesis in two stages**. *Int Orthop* 1995, **19**(3):151-156.

A146. Lux PS, Martin JW, Whiteside LA: **Reinfusion of whole blood after revision surgery for infected total hip and knee arthroplasties**. *J Arthroplasty* 1993, **8**(2):125-128.

A147. Sendi P, Rohrbach M, Graber P, Frei R, Ochsner PE, Zimmerli W: **Staphylococcus aureus small colony variants in prosthetic joint infection**. *Clin Infect Dis* 2006, **43**(8):961-967.

A148. Virolainen P, Lahteenmaki H, Hiltunen A, Sipola E, Meurman O, Nelimarkka O: **The reliability of diagnosis of infection during revision arthroplasties**. *Scand J Surg* 2002, **91**(2):178-181.

A149. Dale H, Hallan G, Espehaug B, Havelin LI, Engesaeter LB: **Increasing risk of revision due to deep infection after hip arthroplasty**. *Acta Orthop* 2009, **80**(6):639-645.

A150. Murray WR: **Use of antibiotic-containing bone cement**. *Clin Orthop Relat Res* 1984, **190**:89-95.

A151. Chen CE, Wang JW, Juhn RJ: **Total hip arthroplasty for primary septic arthritis of the hip in adults**. *Int Orthop* 2008, **32**(5):573-580.

A152. Hunter G: **The results of reinsertion of a total hip prosthesis after sepsis**. *J Bone Joint Surg* 1979, **61-B**(4):422-423.

A153. Kilgus DJ, Howe DJ, Strang A: **Results of periprosthetic hip and knee infections caused by resistant bacteria**. *Clin Orthop Relat Res* 2002, **404**:116-124.

A154. Masri BA, Duncan CP, Beauchamp CP: **Long-term elution of antibiotics from bone-cement: an in vivo study using the prosthesis of antibiotic-loaded acrylic cement (PROSTALAC) system**. *J Arthroplasty* 1998, **13**(3):331-338.

A155. McPherson EJ, Woodson C, Holtom P, Roidis N, Shufelt C, Patzakis M: **Periprosthetic total hip infection: outcomes using a staging system**. *Clin Orthop Relat Res* 2002(403):8-15.

A156. Ong KL, Kurtz SM, Lau E, Bozic KJ, Berry DJ, Parvizi J: **Prosthetic joint infection risk after total hip arthroplasty in the Medicare population**. *J Arthroplasty* 2009, **24**(Suppl 6 ):105-109.

A157. Wentworth SJ, Masri BA, Duncan CP, Southworth CB: **Hip prosthesis of antibiotic-loaded acrylic cement for the treatment of infections following total hip arthroplasty**. *J Bone Joint Surg* 2002, **84-A**:123-128.

A158. Goulet JA, Pellicci PM, Brause BD, Salvati EM: **Prolonged suppression of infection in total hip arthroplasty**. *J Arthroplasty* 1988, **3**(2):109-116.

A159. Herzog R, Morscher E: **Treatment of infected total prosthesis arthroplasty of the hip joint**. *Orthopade* 1995, **24**(4):326-334.

A160. Jupiter JB, Karchmer AW, Lowell JD, Harris WH: **Total hip-arthroplasty in the treatment of adult hips with current or quiescent sepsis**. *J Bone Joint Surg* 1981, **63-A**(2):194-200.

A161. Talbott RD, Glassburn AR, Nelson JP, McElhinney JP, Greenberg RL: **Implantation of total hip arthroplasty after known deep infection**. *Orthop Trans* 1980, **4**:97.

A162. Durbhakula SM, Czajka J, Fuchs MD, Uhl RL: **Spacer endoprosthesis for the treatment of infected total hip arthroplasty**. *J Arthroplasty* 2004, **19**(6):760-767.

A163. Siegel A, Frommelt L, Runde W, Engelbrecht E: **Primary arthroplasty of infected hips and knees in special cases using antibiotic-loaded bone-cement for fixation**. *J Arthroplasty* 2001, **16**(8 Suppl 1):145-149.

A164. Pagnano MW, Trousdale RT, Hanssen AD: **Outcome after reinfection following reimplantation hip arthroplasty**. *Clin Orthop Relat Res* 1997(338):192-204.

A165. Engelbrecht E, Siegel A, Kappus M: **Total hip endoprosthesis following resection arthroplasty**. *Orthopade* 1995, **24**(4):344-352.

A166. Parvizi J, Ghanem E, Azzam K, Davis E, Jaberi F, Hozack W: **Periprosthetic infection : Are current treatment strategies adequate?** *Acta Orthop Belgica* 2008, **74**:793-800.

A167. Lai KA, Yang CY, Lin RM, Jou IM, Lin CJ: **Cementless reimplantation of hydroxyapatite-coated total hips after periprosthetic infections**. *J Formos Med Assoc* 1996, **95**(6):452-457.

A168. Berlusconi M, Molinari G, Tripepi P, Ceroni RG: **Personal experience in revision of infected total hip arthroplasty**. *Minerva Ortopedica e Traumatologica* 1998, **49**(12):459-465.
